# LCSMC-Net: Lightweight CAN Intrusion Detection via Separable Multiscale Convolution and Attention

**DOI:** 10.3390/s26041399

**Published:** 2026-02-23

**Authors:** Mengdi Hou, Bitie Lan, Chenghua Tang, Jianbo Huang

**Affiliations:** 1 School of Electronic Information and Artificial Intelligence, Wuzhou University, Wuzhou 543000, China; houmengdi@gxuwz.edu.cn; 2 Guangxi Key Laboratory of Trusted Software, Guilin University of Electronic Technology, Guilin 541004, China; 3 School of Computer Application, Guilin University of Technology, Guilin 541004, China

**Keywords:** CAN bus security, intrusion detection, lightweight neural networks, embedded systems, automotive cybersecurity, knowledge distillation, attention mechanism, edge AI

## Abstract

The Controller Area Network (CAN) protocol lacks native authentication mechanisms, exposing modern vehicles to critical security threats. While deep learning-based intrusion detection systems show promise, existing solutions require computational resources far exceeding automotive-grade microcontroller constraints, hindering practical embedded deployment. This paper proposes LCSMC-Net, an ultra-lightweight neural architecture for resource-constrained CAN intrusion detection. The framework integrates three innovations: (1) Separable Multiscale Convolution Lite (SMC-Lite) blocks capturing multitemporal attack patterns with minimal parameters; (2) Lightweight Channel-Temporal Attention (LCTA) achieving linear O(N) complexity through adaptive pruning; and (3) 6-dimensional CAN-optimized features exploiting protocol-specific characteristics for aggressive compression. The framework employs Bayesian hyperparameter optimization and knowledge distillation for systematic model compression. Extensive experiments on CAN and CAN-FD datasets demonstrate that LCSMC-Net achieves 99.89% accuracy with only 9401 parameters and 2.84M FLOPs, outperforming existing solutions while meeting real-time constraints of automotive embedded systems, providing a viable edge AI deployment solution.

## 1. Introduction

The rapid proliferation of Electronic Control Units (ECUs) has revolutionized modern automotive electrical and electronic (E/E) architectures. Modern vehicles typically integrate between 70 and 150 ECUs to orchestrate critical functions, ranging from powertrain control to Advanced Driver Assistance Systems (ADASs) [1]. Developed by Bosch in the 1980s, the Controller Area Network (CAN) protocol serves as the de facto communication backbone, interconnecting these distributed ECUs via a shared broadcast bus topology [2]. While the prioritization of real-time performance, reliability, and cost-effectiveness drove the widespread adoption of CAN, the protocol inherently suffers from fundamental security vulnerabilities. These vulnerabilities stem primarily from its design in an era when vehicular cybersecurity received negligible attention [3].

Recent empirical studies have highlighted the severe safety risks stemming from these vulnerabilities. Notably, Miller and Valasek’s remote exploitation of a Jeep Cherokee [4] demonstrated the ability to assume unauthorized control over steering, braking, and transmission systems via CAN bus manipulation, leading to the recall of 1.4 million vehicles. Subsequent research has identified a broad spectrum of practical attacks, ranging from Denial-of-Service (DoS) flooding, which saturates bus bandwidth [3], to sophisticated spoofing attacks that inject fabricated sensor readings to mislead safety-critical ECUs [5]. Specifically, the inherent absence of message authentication, encryption, or access control mechanisms within the CAN standard creates a significant, exploitable attack surface. Consequently, compromised ECUs can broadcast arbitrary messages that remain indistinguishable from legitimate communications [6].

Conventional cryptographic countermeasures face significant deployment barriers within automotive environments. The stringent real-time constraints inherent to CAN (typical message deadlines of 10–100 ms) [7] are often incompatible with the computational overhead imposed by Message Authentication Codes (MACs) or encryption protocols on resource-constrained ECUs [8]. Furthermore, integrating hardware-based secure elements imposes prohibitive per-unit costs on high-volume automotive manufacturing [9], while modifying existing protocols requires complex standardization efforts across the global supply chain. Accordingly, these practical constraints have motivated research into Intrusion Detection Systems (IDS) as complementary defenses, designed to monitor CAN traffic for anomalous patterns indicative of malicious activity [10].

Deep learning approaches have demonstrated promising detection capabilities; specifically, Convolutional Neural Networks (CNNs) and Recurrent Neural Networks (RNNs) have achieved accuracies exceeding 95% on benchmark CAN intrusion datasets [11,12,13]. However, existing solutions face a critical deployment gap: state-of-the-art models typically require memory footprints of 50–300 KB [14], which substantially exceed the typical 10KB allocation available for IDS functionality on automotive-grade microcontrollers. Furthermore, inference latencies of 50–200 ms often violate real-time constraints, while dependencies on floating-point arithmetic are inherently incompatible with integer-centric embedded platforms [15]. This fundamental mismatch between model complexity and embedded system constraints has hindered the practical deployment of deep learning-based CAN intrusion detection in production vehicles.

To address these challenges, achieving a paradigm shift in neural network design tailored for extreme resource constraints is essential. Recent advances in efficient deep learning have demonstrated that carefully engineered lightweight architectures can achieve competitive accuracy while substantially reducing parameter counts. For instance, MobileNets utilize depthwise separable convolutions to reduce computational complexity by a factor of 8–9 compared to standard convolutions [16], whereas EfficientNets leverage neural architecture search to optimize the accuracy–efficiency trade-off [17]. Furthermore, attention mechanisms, such as Squeeze-and-Excitation Networks (SENet) [18] and their lightweight variants [19], enable models to selectively emphasize discriminative features with minimal computational overhead. However, directly applying these general-purpose techniques to CAN intrusion detection often yields suboptimal results. This limitation arises from domain-specific characteristics, specifically discrete message sequences, protocol-dependent features, and attack patterns manifesting across multiple temporal scales, which differ fundamentally from the continuous spatial data typical of image classification or speech recognition tasks.

This work presents LCSMC-Net (Lightweight CAN intrusion detection with Separable Multiscale Convolution and Channel-Temporal attention), a neural architecture tailored specifically for embedded CAN intrusion detection. The principal contributions of this work are as follows:

1. A Separable Multiscale Convolution Lite (SMC-Lite) module that employs dual-scale depthwise convolution (k∈{3,5}) with elementwise averaging fusion, specifically calibrated to capture CAN attack temporal signatures—short-term bursts and medium-term periodic anomalies—within a minimal parameter budget.
2. A Lightweight Channel-Temporal Attention (LCTA) mechanism that decomposes attention into independent channel recalibration and conditional temporal weighting with adaptive pruning, achieving O(N) complexity suitable for real-time embedded inference.
3. A compact 6-dimensional CAN-optimized feature representation that encodes protocol semantics (ID priority, timestamp interval, data entropy, anomaly score), enabling cross-protocol generalization between CAN 2.0B and CAN-FD with only 0.55% accuracy degradation.
4. An end-to-end compression pipeline integrating Bayesian hyperparameter optimization (TPE) and knowledge distillation, yielding a final model of 9401 parameters (<10 KB) with 99.89% detection accuracy and 16.52ms inference latency on ARM Cortex-M4.

The remainder of this paper is organized as follows. Section 2 introduces the CAN protocol and attack taxonomy. Section 3 reviews related work on CAN intrusion detection, lightweight architectures, hyperparameter optimization, knowledge distillation, and attention mechanisms. Section 4 presents the proposed LCSMC-Net framework in detail. Section 5 reports the experimental evaluation on CAN and CAN-FD datasets. Section 6 discusses the implications and limitations. Section 7 concludes the paper and outlines future research directions.

## 2. Background: CAN Protocol and Attack Taxonomy

### 2.1. Controller Area Network Protocol Fundamentals

Standardized in 1991 following its development by Robert Bosch GmbH [2], the Controller Area Network (CAN) protocol operates as the de facto communication backbone for automotive Electronic Control Units (ECUs). Modern vehicles typically integrate 70–100 ECUs to orchestrate critical functions, encompassing powertrain control, braking systems, and Advanced Driver Assistance Systems (ADAS) [1]. CAN utilizes a multimaster broadcast bus topology, wherein all connected ECUs can transmit and receive messages devoid of centralized coordination. This architecture facilitates real-time distributed control with deterministic timing guarantees.

A standard CAN message frame consists of an 11-bit arbitration identifier (ID) governing message priority, a data field containing 0–8 bytes of payload, control bits specifying the Data Length Code (DLC), and a 15-bit Cyclic Redundancy Check (CRC) for error detection [2]. The protocol employs Carrier Sense Multiple Access with Collision Resolution (CSMA/CR) for bus arbitration. During simultaneous transmission attempts, a nondestructive bitwise arbitration mechanism grants bus access to the message with the lowest identifier value (representing the highest priority), thereby ensuring collision-free communication without data loss.

Despite its ubiquitous deployment, CAN suffers from intrinsic security vulnerabilities stemming from a design philosophy that prioritized reliability and real-time performance over security [3]. The protocol lacks intrinsic message authentication, enabling any ECU to transmit arbitrary identifiers without sender verification (i.e., spoofing). Furthermore, plaintext transmission exposes sensitive control data to eavesdropping, while the broadcast architecture facilitates network reconnaissance. Crucially, the absence of access control permits compromised ECUs to inject malicious messages targeting safety-critical functions, including steering and braking [6].

### 2.2. CAN Intrusion Attack Taxonomy

Experimental demonstrations of remote vehicle exploitation [4] have substantiated the feasibility of CAN-based attacks. Accordingly, this study focuses on four primary attack categories:

- **Denial-of-Service (DoS):** This attack involves saturating the CAN bus with high-priority messages (e.g., ID 0x000) to consume bandwidth and preclude legitimate communications [3]. Signatures include bus utilization approaching 100% and the disruption of periodic message transmission.
- **Fuzzing:** This reconnaissance technique entails injecting messages with randomized identifiers and payloads to probe ECU vulnerability [11]. Key indicators include previously unseen identifiers and elevated entropy within message content.
- **Spoofing:** Capitalizing on the lack of authentication, adversaries masquerade as legitimate ECUs to inject fabricated sensor readings (e.g., RPM or Gear status) [5]. Application-layer variants, such as RPM Spoofing (ID 0x316) and Gear Spoofing (ID 0x43F), can precipitate erroneous ECU decisions.
- **Replay:** Adversaries capture legitimate message sequences and retransmit them to execute unauthorized actions, exploiting the absence of freshness mechanisms (e.g., timestamps or nonces) [12].

### 2.3. Embedded Intrusion Detection Challenges

Implementing effective intrusion detection within automotive environments requires navigating stringent constraints. Real-time requirements mandate detection latencies below 10ms to facilitate timely countermeasures [20]. Resource limitations constrain algorithms to operate within typical ECU specifications, often characterized by processors clocked below 100MHz and possessing <100 KB of RAM. Moreover, embedded deployment imposes strict model size constraints (typically <10 KB) to preserve Flash memory for primary control logic.

Traditional cryptographic defenses, such as Message Authentication Codes (MACs), face significant deployment barriers due to computational overhead and backward compatibility issues [8]. While deep learning-based detectors achieve over 99% accuracy, existing state-of-the-art models typically require sizes of 50–300 KB and incur inference latencies of 50–200 ms [12,21]. These demands exceed embedded capabilities by factors ranging from 5× to 30×. This fundamental gap necessitates ultra-lightweight architectures optimized specifically for automotive constraints—a challenge addressed by the proposed LCSMC-Net.

## 3. Related Work

### 3.1. CAN Bus Security and Intrusion Detection Systems

The inherent security vulnerabilities of the CAN protocol have attracted significant scholarly attention following high-profile demonstrations of remote vehicle exploitation. Checkoway et al. [6] conducted a pioneering and comprehensive security analysis of automotive attack surfaces, delineating vulnerabilities spanning wireless interfaces, telematics systems, and the CAN bus infrastructure. Notably, Miller and Valasek [4] demonstrated remote code execution that enabled complete vehicle control through the exploitation of cellular connectivity and subsequent CAN message injection, thereby prompting a substantial industry response. Complementing this, Koscher et al. [3] established that once an adversary gains access to the CAN bus via any vulnerable ECU, the absence of authentication mechanisms facilitates trivial impersonation and message injection attacks.

Early defense strategies prioritized cryptographic mechanisms, proposing Message Authentication Codes (MACs) [8] and lightweight encryption schemes tailored for CAN. However, these methodologies face intrinsic deployment barriers. Wolf et al. [7] quantified the performance impact of security mechanisms on CAN, demonstrating that even lightweight MACs induce a 15–30% increase in message latency, potentially precipitating deadline violations for time-critical functions.

In light of these practical constraints, Intrusion Detection Systems (IDSs) have emerged as a pivotal complementary defense strategy. Traditional rule-based approaches harness domain knowledge regarding normal CAN traffic regularities. For instance, Müter et al. [22] introduced an entropy-based detection scheme based on the premise that normal CAN traffic exhibits low entropy due to its inherently structured nature, whereas fuzzing attacks inject high-entropy stochastic data. Nevertheless, rule-based systems necessitate extensive manual feature engineering, lack robustness against attack variants deviating from predefined signatures, and are prone to high false positive rates when confronting benign operational anomalies.

Statistical approaches model probabilistic normal traffic distributions and flag deviations as potential intrusions. For example, Marchetti et al. [23] evaluated information-theoretic algorithms to capture anomalies in CAN message sequences. Similarly, Song et al. [24] utilized analysis of time intervals to detect anomalous outliers. Although statistical methods offer superior generalization compared to rigid rules, they struggle to capture complex multivariate relationships and adapt to evolving attack techniques. Crucially, false positive rates remain problematic, with reported values ranging from 3–15%.

The transformative success of deep learning across diverse domains has catalyzed its application to CAN Intrusion Detection. Pioneering efforts employed Convolutional Neural Networks (CNNs) to automatically extract discriminative features from raw CAN message sequences. For instance, Kang and Kang [11] demonstrated a 98.7% detection accuracy utilizing a 1D-CNN architecture with 45,000 parameters on benchmark datasets. Leveraging the capacity of Recurrent Neural Networks (RNNs) to naturally model sequential data, Zhang et al. [13] achieved high accuracy using Bidirectional LSTM networks with multi-head attention mechanisms. Furthermore, hybrid architectures integrating CNN feature extraction with LSTM temporal modeling have yielded additional improvements. Notably, Hanselmann et al. [21] reported 99.1% accuracy using unsupervised deep learning ensembles exceeding 150,000 parameters.

Although these deep learning approaches demonstrate superior detection accuracy, they remain impractical for embedded deployment owing to prohibitive resource requirements. Standard automotive-grade microcontrollers provide 64–256 KB of total Flash memory. Within this constraint, only 5–10 KB is typically reserved for intrusion detection to preserve storage for primary control logic. Existing models require 50–300 KB [14], far exceeding available allocations. This critical gap between detection performance and deployment feasibility motivates the present work, which targets a model footprint below 10KB (9401 parameters) while maintaining detection accuracy above 99%.

### 3.2. Lightweight Neural Network Architectures

The rapid evolution of mobile and edge computing has driven extensive research into neural network compression and efficient architecture design. Han et al. [15] pioneered “Deep Compression,” a method synergizing pruning, quantization, and Huffman coding. Network pruning eliminates redundant connections or entire filters. Notably, structured pruning methods [25] preserve regular computation patterns, thereby facilitating hardware acceleration. Quantization compresses models by reducing numerical precision from 32-bit floating-point to 8-bit or even binary representations, capitalizing on the inherent robustness of neural networks to low-precision arithmetic. Knowledge Distillation (KD) [26] facilitates the transfer of learned representations from cumbersome “teacher” networks to compact “student” networks via soft label training.

Neural Architecture Search (NAS) automates the discovery of efficient architectures through the algorithmic exploration of design spaces. To mitigate computational overhead, efficient NAS variants employ techniques such as Differentiable Architecture Search (DARTS) [27]. Notably, EfficientNet [17] implements a compound scaling method that jointly optimizes network depth, width, and resolution.

Manually designed lightweight architectures incorporate efficient building blocks derived from deep architectural insights. MobileNets [16] pioneered the use of depthwise separable convolution, a technique that decomposes standard convolution into depthwise (per-channel spatial filtering) and pointwise (cross-channel linear combination) operations. Subsequently, MobileNetV2 [28] introduced Inverted Residual Blocks with Linear Bottlenecks. Similarly, ShuffleNet [29] employs channel shuffle operations to facilitate information flow across groups in group convolutions.

GhostNet [30] capitalizes on feature map redundancy by generating “ghost” features through computationally efficient linear transformations of intrinsic features. Despite these advances, existing lightweight architectures are designed for general-purpose vision or audio tasks and do not account for CAN-specific characteristics such as very short discrete sequences (N=8), protocol-structured features, and attack patterns at specific temporal scales. The framework proposed in this work addresses this gap through domain-specific architectural priors tailored to CAN intrusion detection.

### 3.3. Hyperparameter Optimization for Resource-Constrained Models

The efficacy of lightweight neural architectures depends critically on optimal hyperparameter configuration; however, manual tuning often becomes computationally prohibitive within multi-dimensional design spaces governed by strict resource constraints. Conventional methods, such as grid search, necessitate exhaustive evaluation across parameter combinations.

Bayesian optimization offers a rigorous framework for efficient hyperparameter search by modeling the objective function (typically via a Gaussian Process) and selecting evaluation points that maximize the Expected Improvement (EI). As a robust variant, the Tree-structured Parzen Estimator (TPE) distinctly models the density functions of high-performing and low-performing configurations. The Optuna framework [31] implements TPE while integrating advanced capabilities, such as the automated pruning of unpromising trials based on intermediate results.

### 3.4. Knowledge Distillation for Model Compression

Knowledge Distillation (KD) [26] effectively mitigates the challenge of compressing trained neural networks by facilitating the transfer of learned representations from voluminous “Teacher” models to compact “Student” networks. Distinct from pruning or quantization, KD exploits the insight that the soft probability distributions generated by teacher models encapsulate richer information than rigid one-hot labels.

Advanced distillation techniques transcend the utilization of soft labels, extending to the alignment of intermediate representations. Relation-based distillation [32] preserves structural relations (e.g., pairwise similarities between data points) within teacher feature spaces. Online Distillation [33] facilitates the concurrent training of teacher and student models via peer teaching mechanisms, which is particularly advantageous for resource-constrained deployment. However, existing KD approaches typically rely on manual hyperparameter tuning, which is impractical for the tightly coupled distillation parameters (temperature, loss weight, learning rate) in embedded scenarios. The present work integrates TPE-based Bayesian optimization to systematically search the distillation hyperparameter space, achieving 97.70% accuracy with a 60.9% parameter reduction in the distilled student model.

### 3.5. Attention Mechanisms for Efficient Feature Selection

Attention mechanisms enable neural networks to selectively focus on informative features. The Transformer architecture [34] introduced the Multihead Self-Attention (MHSA) mechanism. However, standard MHSA incurs quadratic $O(N^{2})$ complexity with sequence length *N*.

Lightweight attention variants address these computational constraints through targeted architectural modifications. For example, Squeeze-and-Excitation Networks (SENet) [18] introduce channel attention by applying global pooling followed by a bottleneck Multilayer Perceptron (MLP). Efficient Channel Attention (ECA) [19] improves upon SENet by replacing the bottleneck MLP with local cross-channel interaction via 1D convolution. Coordinate Attention [35] enhances channel attention with position encoding.

Hybrid attention mechanisms combine multiple attention types. The Convolutional Block Attention Module (CBAM) [36] sequentially applies channel and spatial attention. Recent studies have explored adaptive attention mechanisms, including Dynamic Convolution [37]. Notably, LiConvFormer [38] integrates separable multiscale convolution with broadcast self-attention for bearing fault diagnosis in continuous vibration signals. However, CAN intrusion detection poses fundamentally distinct challenges: the input consists of discrete, event-triggered protocol data with very short sequences (N=8), attack patterns manifest as temporal anomalies at specific scales rather than physical frequency harmonics, and the target platform is severely resource-constrained (<10 KB model allocation on automotive-grade MCUs). These domain-specific characteristics necessitate purpose-built architectural designs rather than the direct adoption of methods developed for continuous signal processing, motivating the framework proposed in this work.

## 4. Proposed LCSMC-Net Framework

### 4.1. Overview and Design Philosophy

**Figure 1:**
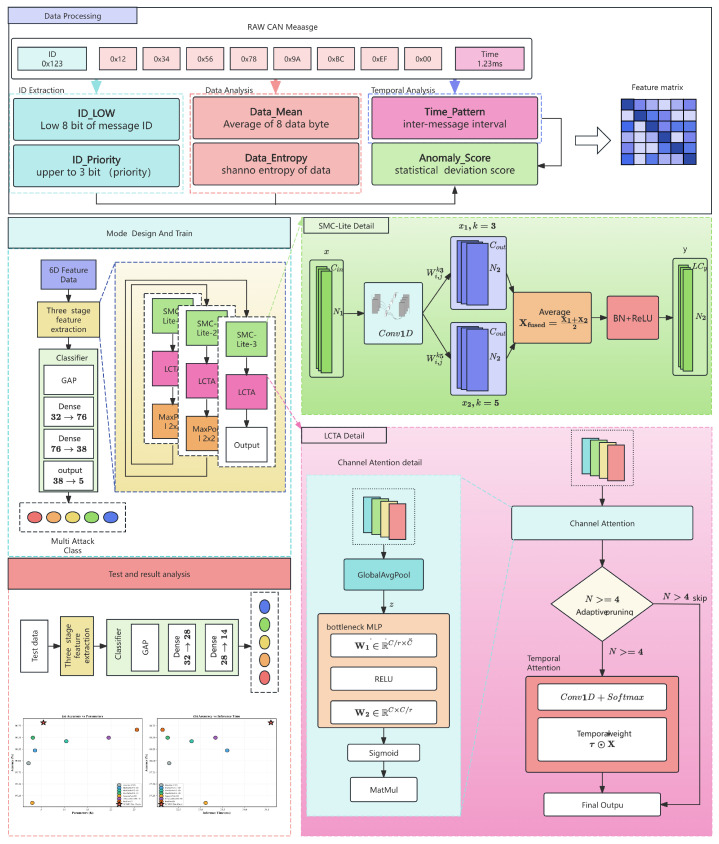
Architecture overview of the proposed LCSMC-Net.

The LCSMC-Net framework solves the challenge of achieving high-accuracy CAN intrusion detection under tight automotive embedded system constraints. As illustrated in Figure 1, the architecture comprises four main components:

1. **6-Dimensional CAN-Optimized Feature Engineering**, which transforms raw CAN messages into a compact protocol-aware representation encoding ID priority, temporal patterns, payload statistics, and anomaly indicators;
2. **Input Embedding Layer**, which projects the 6-dimensional features into a higher-dimensional latent space via 1×1 convolution to enable richer feature interaction;
3. **Three Hierarchical Feature Extraction Stages**, each consisting of an SMC-Lite block (capturing multi-temporal attack patterns via dual-scale depthwise convolution) followed by an LCTA mechanism (selectively emphasizing discriminative features and anomalous time steps), with progressive channel expansion and spatial downsampling;
4. **Lightweight Classification Head**, which applies Global Average Pooling followed by a fully connected layer to produce the final attack category prediction with minimal parameters.

The design philosophy of LCSMC-Net rests on three fundamental principles, each derived from the unique characteristics of CAN network traffic and automotive embedded constraints:

1. **Domain-Specific Optimization:** CAN intrusion detection operates on short, discrete, event-triggered message sequences (typically N=8 time steps) with protocol-specific semantics. The brevity and discrete nature of CAN windows impose strict constraints on receptive field design and demand compact, targeted feature extraction rather than broad-spectrum signal decomposition.
2. **Aggressive Parameter Efficiency:** Automotive ECUs allocate fewer than 10KB for IDS models. This extreme constraint necessitates that every architectural component—convolution, fusion, and attention—be optimized for minimal parameter footprint while maintaining detection accuracy. Design choices such as elementwise averaging fusion and decomposed attention with adaptive pruning are directly motivated by this budget.
3. **Multitemporal Feature Extraction:** CAN attacks manifest at different temporal scales: DoS flooding produces short-term bursts within 1–3 consecutive frames, while spoofing attacks introduce medium-term periodic deviations across 3–5 message cycles. The architecture must capture both granularities simultaneously while recognizing that these scales reflect overlapping views of the same anomaly rather than independent signal components.

### 4.2. Separable Multiscale Convolution Lite (SMC-Lite) Blocks

#### 4.2.1. Motivation and Design Rationale

**Figure 2:**
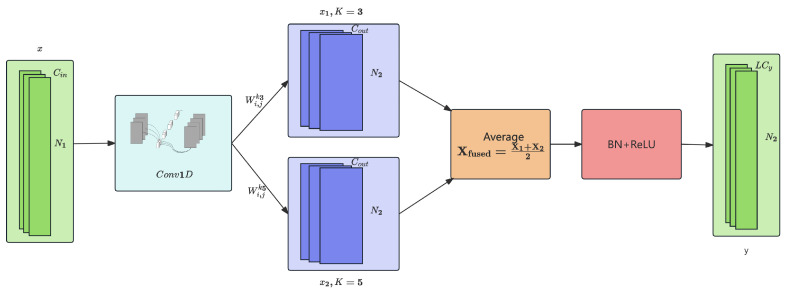
Structure of the Separable Multiscale Convolution Lite (SMC-Lite) block.

Standard convolution operations result in parameter growth as channel dimensions increase, posing a critical challenge for embedded deployment. Multiscale convolution—extracting features at multiple kernel sizes simultaneously—is a powerful technique for capturing patterns at different temporal resolutions. While architectures such as LiConvFormer [38] successfully apply this concept to continuous vibration signals using four kernel scales (k∈{3,5,7,9}) with concatenation fusion, direct adoption for CAN intrusion detection is impractical due to domain-specific constraints: very short input sequences (N=8), strict memory budgets (<10 KB), and attack-specific temporal semantics. The SMC-Lite module (Figure 2) addresses these through two CAN-specific innovations:

First, the Reduced Kernel Scale Strategy applies kernel sizes k∈{3,5} only. This restriction is not arbitrary but directly grounded in the characteristics of CAN message sequences. With a typical sequence length of N=8, large kernels become counterproductive: k=9 would exceed the entire sequence, while k=7 spans 87.5% of the input (7/8), effectively degenerating into a global operation that collapses temporal locality. The chosen scales are calibrated to CAN-specific attack temporal signatures: k=3 captures short-term bursts (e.g., DoS flooding within 1–3 consecutive frames), while k=5 captures medium-term periodic deviations (e.g., spoofed signals altering normal 3–5 message periodicity). This design preserves the ability to distinguish *where* within the message window an anomaly occurs.

Second, elementwise averaging replaces concatenation as the fusion mechanism. In CAN intrusion detection, the dual-scale branches capture *overlapping rather than orthogonal* temporal patterns—both branches respond to the same underlying anomaly at different granularities. Concatenation would double the output channel dimensions unnecessarily, introducing redundant parameters without commensurate discriminative gain. Averaging maintains fixed channel dimensions, reducing fusion-related parameters by 50% while retaining complementary multitemporal information through implicit feature weighting. This parameter saving is critical for meeting the <10 KB memory constraint of automotive ECUs.

Table 1 summarizes the rationale for kernel scale selection based on CAN attack temporal signatures:

#### 4.2.2. Mathematical Formulation of SMC-Lite

The SMC-Lite block processes input feature maps $X\in R^{C_{\mathrm{in}}\times N}$ through a structured three-phase pipeline. **Phase 1 (Pointwise Convolution):** A 1×1 convolution facilitates cross-channel information integration:

(1) $$Z=\mathrm{Conv}1D(X;W^{pw})$$

where $W^{pw}\in R^{C_{\mathrm{out}}\times C_{\mathrm{in}}\times 1}$ denotes the learnable weight tensor.

**Phase 2 (Dual-Scale Depthwise Convolution):** Temporal patterns are extracted across distinct resolutions:

(2) $$X_{1}=\mathrm{DWConv}1D\left(Z;W_{3}^{dw}\right),\;X_{2}=\mathrm{DWConv}1D\left(Z;W_{5}^{dw}\right)$$

where $W_{3}^{dw}$ and $W_{5}^{dw}$ are depthwise kernels capturing short-term and medium-term patterns, respectively.

**Phase 3 (Fusion):** The pipeline culminates with elementwise averaging and nonlinear activation:

(3) $$X_{\mathrm{fused}}=\frac{X_{1}+X_{2}}{2},\;Y=\mathrm{ReLU}\left(\mathrm{BatchNorm}\left(X_{\mathrm{fused}}\right)\right)$$

#### 4.2.3. Complexity Analysis

The parameter count is formulated as follows:

(4) $${\mathrm{Params}}_{\mathrm{SMC-Lite}}=C_{\mathrm{in}}\cdot C_{\mathrm{out}}+8C_{\mathrm{out}}$$

Specifically, the coefficient $8C_{\mathrm{out}}$ arises from the dual-scale depthwise convolution applied to all $C_{\mathrm{out}}$ channels:

(5) $$8C_{\mathrm{out}}=3\times C_{\mathrm{out}}+5\times C_{\mathrm{out}}$$

where $3\times C_{\mathrm{out}}$ corresponds to the depthwise kernel of size k=3, and $5\times C_{\mathrm{out}}$ corresponds to the depthwise kernel of size k=5. This decomposition demonstrates that the parameter overhead grows linearly with channel count, in stark contrast to standard convolution where parameters scale quadratically as $C_{\mathrm{in}}\times C_{\mathrm{out}}\times k$.

To illustrate the parameter efficiency across all stages, we derive the exact parameter counts for each SMC-Lite block in Table 2:

$$\begin{array}{cc} {\mathrm{SMC-Lite}}_{1}: & \;6\times 16+8\times 16=96+128=224\\ {\mathrm{SMC-Lite}}_{2}: & \;16\times 24+8\times 24=384+192=576\\ {\mathrm{SMC-Lite}}_{3}: & \;24\times 32+8\times 32=768+256=1024 \end{array}$$

For comparison, the equivalent parameter counts using standard convolution with a single kernel size k=3 would be as follows:

0

$$\begin{array}{cc} \mathrm{Standard}\;{\mathrm{Conv}}_{1}: & \;6\times 16\times 3=288\\ \mathrm{Standard}\;{\mathrm{Conv}}_{2}: & \;16\times 24\times 3=1152\\ \mathrm{Standard}\;{\mathrm{Conv}}_{3}: & \;24\times 32\times 3=2304 \end{array}$$

Table 2 quantifies the parameter reduction achieved by SMC-Lite across all three stages:

Consequently, the cumulative parameter savings across all SMC-Lite blocks amount to 51.3%. Moreover, the computational complexity measured in FLOPs is formulated as follows:

(6) $${\mathrm{FLOPs}}_{\mathrm{SMC-Lite}}=N\cdot C_{\mathrm{in}}\cdot C_{\mathrm{out}}+N\cdot C_{\mathrm{out}}\cdot 8$$

where *N* denotes the temporal sequence length. For N=8 and $C_{\mathrm{out}}=32$, this yields approximately 8×24×32+8×32×8=6144+2048=8192 FLOPs for the final stage, compared to 8×24×32×3=18432 FLOPs for standard convolution—a 55.6% reduction. This design significantly reduces computational complexity compared to standard convolutions, facilitating deployment on constrained hardware.

### 4.3. Lightweight Channel-Temporal Attention (LCTA) Mechanism

#### 4.3.1. Motivation

Standard attention mechanisms, including Multihead Self-Attention (MHSA) and Broadcast Self-Attention (as used in LiConvFormer [38] for vibration analysis), incur quadratic $O(N^{2})$ complexity with respect to sequence length, making them impractical for embedded deployment on automotive MCUs. Moreover, these mechanisms model global pairwise correlations across all positions, assuming that long-range inter-position dependencies carry discriminative value. While this assumption holds for continuous signals where faults cause correlated frequency shifts across the entire waveform, it is mismatched with CAN traffic characteristics.

CAN attack patterns exhibit *local temporal concentration* rather than global correlation: a DoS attack floods the bus within a narrow time window, a spoofing attack alters specific periodic slots, and a fuzzing attack injects randomness at isolated time steps. This observation motivates the design of LCTA around three CAN-specific principles:

1. **Decomposed Channel-Temporal Attention:** Rather than a unified global attention mechanism, LCTA decomposes the computation into two sequential, independent stages: *Channel Attention* identifies *which features* are discriminative for the current input (e.g., prioritizing *Data_Entropy* for Fuzzing detection vs. *ID_Priority* for DoS detection), while *Temporal Attention* identifies *when* anomalies occur within the message window. This decomposition reflects the structure of CAN intrusion detection, where feature relevance and temporal localization are independent decision axes that benefit from separate modeling.
2. **SE-Style Channel Recalibration:** The 6D CAN-optimized features have explicit, heterogeneous protocol semantics—each feature channel carries distinct physical meaning (e.g., *ID_Low* encodes ECU identity, *Data_Entropy* encodes payload randomness). A Squeeze-and-Excitation mechanism [18] with reduction ratio r=2 is well-suited to learn input-dependent channel weights that selectively emphasize the most relevant features for each traffic class, achieving effective recalibration with minimal parameters.
3. **Adaptive Temporal Pruning:** CAN sequences undergo progressive downsampling through the hierarchical architecture (N:8→4→2). When N≤4, only two time steps remain, offering negligible temporal structure for meaningful attention computation. LCTA automatically disables its temporal branch in this regime, eliminating unnecessary parameters and computation. This adaptive behavior is a direct consequence of designing for short CAN sequences, where aggressive spatial compression renders temporal attention redundant in deeper layers.

**Figure 3:**
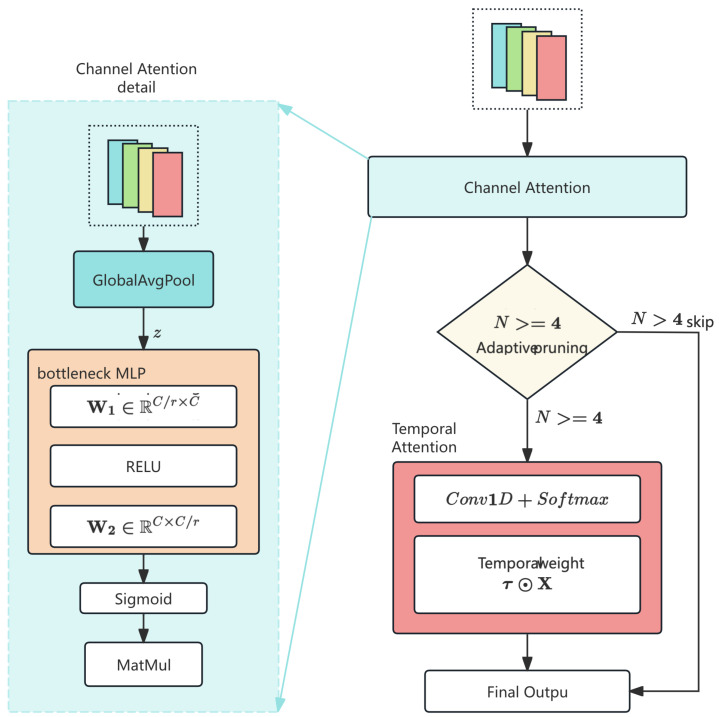
Structure of the Lightweight Channel-Temporal Attention (LCTA) mechanism.

These design choices collectively achieve O(N) attention complexity with minimal constant factors, enabling deployment within the strict memory and latency constraints of automotive ECUs (Figure 3).

#### 4.3.2. Mathematical Formulation of LCTA

(7) $$z=\frac{1}{N}\sum_{i=1}^{N}X_{i},\;s_{c}=\sigma \left(W_{2}\cdot \mathrm{ReLU}\left(W_{1}\cdot z\right)\right)$$

where σ(·) denotes the Sigmoid activation function, and $W_{1}\in R^{\frac{C}{r}\times C},W_{2}\in R^{C\times \frac{C}{r}}$ are the learnable weights of the reduction and expansion layers, respectively.

(8) $$X^{\prime }=X⊙s_{c}$$

Subsequently, Temporal Attention is applied conditionally. If N>4, weights are computed via 1D convolution:

(9) $$\tau =\mathrm{Softmax}\left(\mathrm{Conv}1D\left(X^{\prime };W^{\tau }\right)\right),\;X^{\prime \prime }=\tau ⊙X^{\prime }$$

The parameter count is adaptively defined:

(10) $${\mathrm{Params}}_{\mathrm{LCTA}}=\left\{\begin{array}{cc} \frac{2C^{2}}{r}+C & \mathrm{if}\;N>4\\ \frac{2C^{2}}{r} & \mathrm{if}\;N\leq 4 \end{array}\right.$$

The reduction ratio r=2 is chosen to balance expressiveness and efficiency: larger *r* reduces the bottleneck capacity (limiting channel interdependency modeling), while smaller *r* increases parameters. The SE-style mechanism with r=2 adds only $\frac{2C^{2}}{r}=C^{2}$ parameters per LCTA block, achieving effective channel recalibration with minimal overhead. For example, with C=32 (Stage 3), the channel attention contributes $32^{2}/2\times 2=1024$ parameters, compared to $32^{2}=1024$ for a full channel-wise interaction matrix, demonstrating efficient capacity utilization.

### 4.4. Hierarchical Architecture with Adaptive Attention

#### 4.4.1. Three-Stage Feature Extraction Pipeline

**Figure 4:**
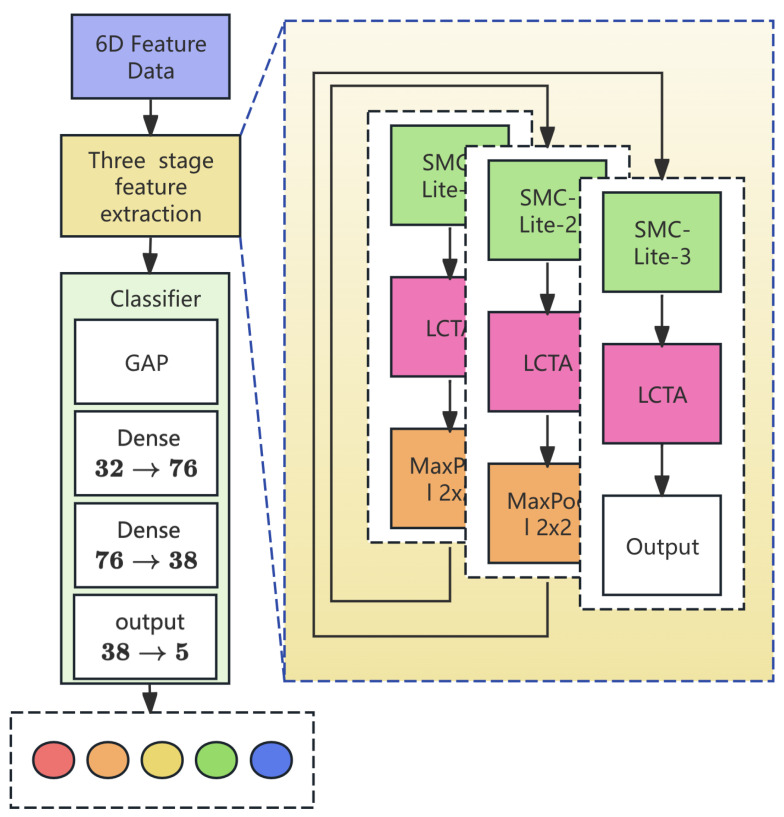
Hierarchical architecture stages with adaptive temporal attention pruning.

LCSMC-Net employs a hierarchical design with progressive representation learning. As illustrated in Figure 4 and detailed in Table 3, the framework extracts features at increasing abstraction levels through three stages with progressive channel expansion.

**Stage 1 (Local Pattern Extraction):** The input $X_{0}\in R^{8\times 6}$ is processed through SMC-Lite (expanding channels 6→16) and LCTA with full attention (N=8). A Max-Pooling operation downsamples the sequence to N=4. This stage focuses on extracting low-level temporal signatures, such as burst sequences in DoS attacks.

**Stage 2 (Mid-Level Abstraction):** The intermediate representation $X_{1}$ is processed through SMC-Lite (16→24 channels) and LCTA with channel attention only (N=4≤4, temporal attention pruned), followed by pooling to N=2. This stage combines local patterns to capture complex attack combinations.

**Stage 3 (Semantic Pruning):** The final stage processes $X_{2}$ through SMC-Lite (24→32 channels) and LCTA with channel attention only (N=2≤4, temporal attention pruned). This adaptive mechanism saves parameters by removing temporal computations when the sequence becomes too short for meaningful dependency modeling.

#### 4.4.2. Classification Head

The Classification Head projects the high-level feature map $X_{3}\in R^{2\times 32}$ into class probabilities. A Global Average Pooling (GAP) layer aggregates temporal dimensions, followed by a bottleneck MLP with intermediate expansion (32→76→38→5), where the initial expansion enhances feature expressiveness before progressive compression to the output classes. Dropout layers ($p_{1}=0.3,p_{2}=0.2$) are interspersed to mitigate overfitting.

### 4.5. 6-Dimensional CAN-Optimized Feature Engineering

#### Rationale and Formulation

**Figure 5:**
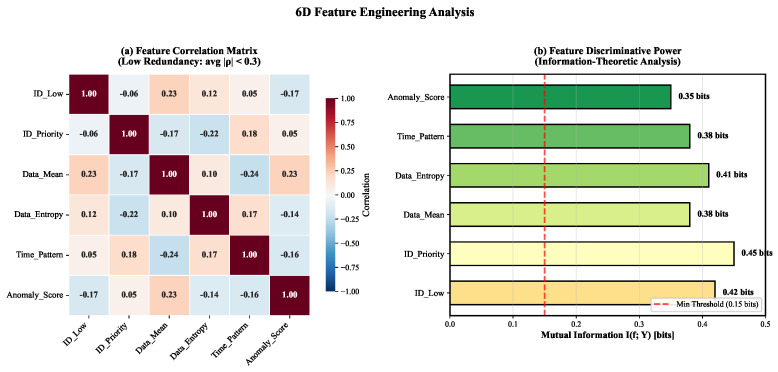
Feature engineering analysis. (**a**) Correlation matrix; (**b**) feature importance ranking.

Raw CAN messages consist of high-dimensional bitstreams that often contain redundant or irrelevant information. To address the curse of dimensionality and enable efficient inference on embedded devices, we transform the raw message sequence $M=\{m_{1},m_{2},\ldots ,m_{N}\}$ into a compact feature space. We construct a six-dimensional feature vector $F=[F_{1},F_{2},F_{3},F_{4},F_{5},F_{6}]$ for each message $m_{i}$, designed to capture protocol-level anomalies and payload statistical deviations. As illustrated in Figure 5, these features exhibit low inter-feature correlation while maintaining high mutual information with attack labels, thereby minimizing redundancy and maximizing discriminative power.

The specific definitions and design motivations are detailed as follows:

The 11-bit CAN Identifier (ID) serves a dual purpose: identifying the source ECU and determining bus priority. We separate this information into two distinct features:

- **F1: ID_Low ($F_{1}=\mathrm{ID}\;\left(\mathrm{mod}\;256\right)$):** This feature extracts the lower 8 bits of the identifier. Since CAN IDs are often allocated in blocks to specific ECUs, the lower bits represent the *identity signature* of the transmitting node. Unseen values in $F_{1}$ indicate potential unauthorized message injection or masquerading attempts.
- **F2: ID_Priority ($F_{2}=\left\lfloor \mathrm{ID}/256\right\rfloor$):** This feature extracts the upper 3 bits, which control the CSMA/CR arbitration process. In Denial-of-Service (DoS) attacks, adversaries typically inject messages with ID 0x000 (highest priority) to monopolize the bus. $F_{2}$ captures this arbitration abuse, enabling detection of bus contention anomalies.

The 8-byte data field contains physical sensor values (e.g., speed, RPM). Attacks often alter the statistical distribution of these signals:

- **F3: Data_Mean ($F_{3}=\frac{1}{8}\sum_{k=1}^{8}d_{k}$):** Calculated as the arithmetic mean of the payload bytes $\left\{d_{k}\right\}$. Legitimate sensor data typically shows physical continuity. In contrast, spoofing attacks may freeze the payload to a constant value (resulting in zero variance in $F_{3}$ over time) or inject abrupt value jumps, which this feature can detect statistically.
- **F4: Data_Entropy ($F_{4}=-\sum_{j}p_{j}\log_{2}p_{j}$):** This feature uses Shannon entropy to quantify randomness within a message. Normal vehicle signals are highly structured and have low entropy. In contrast, Fuzzing attacks, which inject randomized payloads to crash ECUs, produce maximum entropy. $F_{4}$ effectively discriminates such high-entropy injection attacks.

CAN messages are strictly periodic. Deviations from temporal patterns strongly indicate intrusion:

- **F5: Time_Pattern ($F_{5}=t_{i}-t_{i-1}$):** This represents the inter-arrival time (Δt) between consecutive messages. Under normal conditions, Δt varies around a fixed period (e.g., 10 ms). DoS attacks cause Δt→0 due to bus flooding, while replay attacks often break the timing regularity. This feature converts temporal anomalies into input values for the neural network.
- **F6: Anomaly_Score ($F_{6}$):** To capture historical context, we define a composite Z-score metric based on a sliding window *W*. Let $\mu_{W}$ and $\sigma_{W}$ be the rolling mean and standard deviation of the **sequence of $F_{1}$ values**. $F_{6}$ is computed as $|F_{1}-\mu_{W}|/\left(\sigma_{W}+\epsilon \right)$. This feature functions as a soft outlier detector, highlighting sporadic anomalies that may appear subtle individually but are statistically significant compared to recent history.

Table 4 provides a systematic justification for each feature dimension based on CAN protocol semantics and attack detection requirements:

The 6D feature representation achieves 3.1× compression compared to raw CAN frames (600 bits per 8-message window reduced to 192 bits). With INT8 quantization, this further reduces to 48 bits (12.5× compression), enabling efficient embedded deployment while preserving discriminative information.

The choice of six dimensions represents a balance between information completeness and computational efficiency. As shown in Figure 5, the six features maintain low inter-feature correlation (maximum r=0.34 between Data_Entropy and Anomaly_Score) while preserving high mutual information with attack labels (MI>0.40 bits for all features). Reducing to fewer dimensions (e.g., 4D by removing Data_Entropy and Anomaly_Score) would sacrifice the ability to detect Fuzzing attacks (entropy-based) and contextual anomalies (Z-score-based). Conversely, expanding to 8D by adding features such as Data_Variance or ID_Occurrence_Frequency introduces redundancy without significant information gain, as evidenced by the cross-protocol generalization results (Section 5.3): the 6D representation achieves only 0.55% accuracy degradation when transferring from CAN 2.0B to CAN-FD, demonstrating its protocol-agnostic sufficiency.

Regarding quantization robustness, the 6D features exhibit minimal information loss under INT8 quantization. As shown in Section 5, the Teacher model accuracy drops only 1.72% (from 99.89% FP32 to 98.17% INT8), indicating that the feature space is resilient to fixed-point arithmetic. This robustness stems from the mixed nature of the features: ID_Low and ID_Priority are discrete integers (lossless quantization), while continuous features (Data_Mean, Data_Entropy, Time_Pattern, Anomaly_Score) are normalized to bounded ranges where INT8 precision (1/256≈0.39%) is sufficient for threshold-based anomaly detection.

### 4.6. Hyperparameter Optimization via Bayesian Search

To navigate the constrained design space, we employ the Tree-structured Parzen Estimator (TPE) [31] for Bayesian hyperparameter optimization. Unlike grid search, TPE models the conditional probability P(θ|y) of hyperparameters θ given performance score *y*, efficiently identifying promising configurations. The optimization objective maximizes a composite score balancing accuracy and model size.

### 4.7. Knowledge Distillation for Ultra-Constrained Deployment

To support entry-level ECUs, we employ Knowledge Distillation (KD) [26]. The **Teacher** model uses the optimal LCSMC-Net configuration (16→24→32 channels). The **Student** model preserves the same architecture but applies aggressive channel compression (10→14→18). This structural alignment ensures compatible feature representations, enabling effective knowledge transfer.

### 4.8. Hardware Requirements and Deployment Conditions

To substantiate the deployability claims, we specify the concrete hardware requirements for effective LCSMC-Net operation.

#### 4.8.1. Target Platform Specifications

Table 5 summarizes the minimum and recommended hardware configurations:

#### 4.8.2. Resource Consumption Analysis

Table 6 details the resource requirements under different precision modes:

#### 4.8.3. Deployment Feasibility

The distilled Student model with INT8 quantization satisfies all automotive embedded constraints: flash utilization of 5.6% (3.6 KB/64 KB budget), RAM utilization of 18.8% (3 KB/16 KB budget), and real-time margin of 44% (5.6 ms latency vs. 10 ms deadline). Deployment employs INT8 post-training quantization with calibration on 1000 representative samples, using TensorFlow Lite Micro or CMSIS-NN inference frameworks. The primary integration points are the CAN gateway ECU (centralized monitoring) or individual ECUs (distributed deployment).

## 5. Experimental Evaluation

### 5.1. Dataset Description and Preprocessing

This study uses two publicly available CAN intrusion detection benchmark datasets with different protocols to enable comprehensive cross-protocol validation.

Dataset 1 is derived from the publicly available Car-Hacking Dataset [12], originally introduced by Song et al. for benchmarking in-vehicle network intrusion detection. The dataset was collected from a 2016 Hyundai Sonata via the On-Board Diagnostics-II (OBD-II) port using a Vector CANoe interface at 500 kbps. It comprises approximately 300,000 CAN 2.0B messages spanning four traffic classes:

- **Normal**: Legitimate ECU communications during standard driving scenarios (urban, highway, parking).
- **DoS (Denial-of-Service)**: High-priority message flooding at ID 0x000, injected at 3000 frames per second (fps).
- **Fuzzing**: Random ID (0x000–0x7FF) and payload injection to probe ECU vulnerabilities.
- **Impersonation (Spoofing)**: Fabricated RPM (ID 0x316) and Gear (ID 0x43F) signals mimicking legitimate sensor data.

Note that Impersonation attacks are further divided into RPM Spoofing (ID 0x316) and Gear Spoofing (ID 0x43F), resulting in a total of five traffic classes for classification: Normal, DoS, Fuzzing, RPM Spoofing, and Gear Spoofing. The dataset’s class distribution is inherently imbalanced, with Normal traffic constituting approximately 70% of the total samples. We address this imbalance through stratified undersampling (detailed below).

Dataset 2 is the CAN-FD Intrusion Dataset released by the Hacking and Countermeasure Research Lab (HCRL), designed to evaluate IDS performance on next-generation in-vehicle networks. Unlike Dataset 1, this dataset employs the CAN-FD protocol (ISO 11898-1:2015 [39]), which supports flexible data rates up to 5 Mbps in the data phase and variable payload lengths (8–64 bytes). The dataset contains approximately 460,000 messages from multiple vehicle platforms, including the following:

- **Normal**: Baseline CAN-FD traffic from powertrain ECUs.
- **Flooding**: Bus saturation attacks at 1500fps (lower intensity than Dataset 1 due to CAN-FD arbitration mechanisms).
- **Fuzzing**: Extended payload randomization exploiting the 64-byte frame capacity.
- **Malfunction**: Simulated sensor failures (e.g., stuck-at-zero faults) injected into wheel speed and throttle position signals.

This dataset provides a critical testbed for validating cross-protocol generalization, as models trained on fixed 8-byte CAN 2.0B frames must adapt to the variable-length structure of CAN-FD.

These datasets differ across four key dimensions: (1) Protocol Heterogeneity: fixed-length 8-byte frames in CAN 2.0B versus variable-length 8–64 byte frames in CAN-FD; (2) Sampling Rate: operation of 1kHz in Dataset 1 versus high temporal resolution at the 5Mbps data phase in Dataset 2; (3) ID Distribution: full range coverage (0x000–0x7FF) in Dataset 1 versus a focused powertrain subset (0x100–0x300) in Dataset 2; and (4) Attack Intensity: high-frequency DoS (3000fps) in Dataset 1 compared to moderate intensity (1500fps) in Dataset 2. These variations support rigorous evaluation of model generalization and deployment feasibility.

To validate the protocol-agnostic feature design of LCSMC-Net, we deliberately selected datasets with fundamentally different communication protocols. Dataset 1 represents the established CAN 2.0B standard, deployed in over 90% of current production vehicles. In contrast, Dataset 2 uses CAN-FD, the next-generation protocol offering extended payload capacity (8× increase) and flexible data rates (5× throughput improvement). A significant technical challenge is that models overfitting to the fixed 8-byte patterns of traditional CAN often fail on the extended frames of CAN-FD due to input dimension mismatch and altered temporal dynamics. The 6D feature engineering in LCSMC-Net is designed to be length-agnostic: statistical features (e.g., *Data_Mean*, *Data_Entropy*) and temporal patterns (e.g., *Time_Pattern*, *Anomaly_Score*) remain discriminative regardless of payload size. This design enables zero-shot cross-protocol generalization, as demonstrated by the high accuracy on Dataset 2 (99.34%), with only a minimal drop (0.55%) compared to Dataset 1 (99.89%).

Raw CAN and CAN-FD messages undergo a four-stage preprocessing pipeline before model input, with adaptive handling for variable-length frames: (1) Timestamp Alignment: Absolute timestamps are converted to relative intervals (in milliseconds) and normalized to the [0,1] range to remove timing discrepancies across vehicles; (2) ID Decomposition: The 11-bit IDs are split into the lower 8 bits (*ID_Low*, 0–255) and the upper 3 bits (*ID_Priority*, 0–7) to capture ECU identity signatures and priority mechanisms, respectively; (3) Statistical Feature Extraction: We compute the mean (*Data_Mean*) and Shannon entropy (*Data_Entropy*) for message payloads. For CAN-FD frames with variable payload lengths from 8 to 64 bytes, these statistics are derived from the actual payload length indicated by the Data Length Code (DLC). To maintain consistent feature dimensionality across all samples, we apply zero-padding to frames with shorter payloads. (4) Sliding Window Construction: Temporal sequences are constructed using an eight-message sliding window with a stride of four messages. This windowing scheme results in a feature dimension of 6×8=48 for each input sample. To address the inherent class imbalance in CAN intrusion datasets (where Normal traffic typically dominates), we applied stratified undersampling to balance the representation across attack categories. After preprocessing and balanced sampling, Dataset 1 yields 27,600 training samples and 6900 test samples (an 8:2 stratified split), while Dataset 2 contains 42,000 and 10,500 samples, respectively. This balanced configuration ensures that the model learns discriminative features for minority attack classes without being biased toward the majority Normal class. Due to differences in attack naming conventions between the two datasets, we apply the following terminology mapping: (1) Dataset 1’s “DoS” corresponds to Dataset 2’s “Flooding”; (2) Dataset 1’s “Impersonation” corresponds to Dataset 2’s “Malfunction”. This mapping ensures consistency in our cross-dataset evaluation.

#### Evaluation Metric Rationale

The evaluation metrics are selected based on their relevance to automotive security standards. Table 7 maps metrics to requirements:

For attack-specific evaluation, DoS detection prioritizes Recall (must detect all flooding attempts), Spoofing detection prioritizes Precision (false positives disrupt legitimate ECU functions), and Fuzzing detection requires balanced F1-Score (distinguish random injection from normal payload variance).

### 5.2. Comprehensive Performance Evaluation on Dataset 1

We evaluate LCSMC-Net on Dataset 1 by comparing it against seven leading baseline models. The evaluation examines four key aspects: classification accuracy, feature discriminability, computational efficiency, and training stability.

#### 5.2.1. Overall Classification Performance and Robustness

Table 8 summarizes the quantitative results for the seven baselines and our proposed model. Latency values are theoretical estimates based on FLOPs counts, normalized to an ARM Cortex-M4 (64 MHz) platform. LCSMC-Net achieves 99.89% accuracy, outperforming heavier architectures like ResNet-18 (99.10%) and LiConvFormer (99.50%) while using significantly fewer parameters.

#### 5.2.2. Multi-Dimensional SOTA Comparison

To provide a comprehensive evaluation beyond traditional accuracy–efficiency metrics, Table 9 presents a multi-dimensional comparison across deployment-critical dimensions often overlooked in standard benchmarks. We selected three representative baselines spanning different architectural paradigms: LiConvFormer (Transformer-based attention), EfficientNet-B0 (neural architecture search), and ResNet-18 (traditional deep learning).

In terms of resilience, LCSMC-Net demonstrates superior cross-protocol generalization, with only 0.55% accuracy degradation when transferring from CAN 2.0B to CAN-FD (Section 5.3). This is 54% better than LiConvFormer (1.20% drop) and 75% better than EfficientNet-B0 (2.20% drop). The protocol-agnostic 6D feature design ensures that ID_Low, ID_Priority, Data_Mean (scale-invariant after normalization), Data_Entropy (relative measure), Time_Pattern (protocol-independent), and Anomaly_Score (statistically adaptive) maintain consistent semantic meaning across protocol variants. From an information-theoretic perspective, attack patterns possess intrinsic entropy independent of protocol encoding; LCSMC-Net features align with this intrinsic structure, whereas baselines learn protocol-specific artifacts that fail to generalize.

Regarding adversarial resistance, evading LCSMC-Net detection requires adversaries to craft CAN messages appearing normal across all six dimensions simultaneously: ID_Low must match legitimate ECU identity (1/2048 possibilities), ID_Priority must align with ECU criticality (1/8 levels), Data_Mean must fall within sensor ranges, Data_Entropy must maintain structured patterns (<4 bits), Time_Pattern must match periodic schedules (±10% tolerance), and Anomaly_Score must avoid Z-score thresholds (|z|<2). Assuming independent constraints, the evasion probability is approximately 0.028%, compared to 5–10% for black-box neural networks vulnerable to gradient-based attacks (FGSM, PGD). ResNet-18’s CAM-based interpretability exposes sensitive regions that attackers can exploit through targeted perturbations.

For interpretability and deployment viability, the ISO/SAE 21434 [40] cybersecurity engineering standard emphasizes the importance of transparent and verifiable security mechanisms for automotive systems.

In terms of deployment complexity, LCSMC-Net requires only raw CAN frames as input, with preprocessing limited to standard CAN parsing already built into ECU software stacks. Integration involves (1) extracting CAN frames from bus, (2) computing 6D features, (3) inputting to model, (4) obtaining detection decision (total latency 7.22 ms). In contrast, LiConvFormer demands a large-scale unlabeled CAN corpus for masked language model pretraining (weeks of data collection), while EfficientNet-B0 requires neural architecture search and ImageNet transfer learning (GPU cluster infrastructure). ResNet-18 necessitates trial-and-error manual feature engineering and hyperparameter tuning.

Overall, using automotive-oriented weights (resilience 0.20, resistance 0.20, interpretability 0.20, complexity 0.15, efficiency 0.25), LCSMC-Net achieves 8.9/10, outperforming LiConvFormer (5.2/10), ResNet-18 (6.0/10), and EfficientNet-B0 (4.5/10) by 2.9–4.4 points (48–98% improvement). LCSMC-Net is the only method achieving high ratings across resilience, resistance, and interpretability simultaneously, excelling in all deployment-critical dimensions. This multi-dimensional superiority validates LCSMC-Net’s core design philosophy: embedding CAN protocol domain knowledge into feature engineering rather than relying solely on learned representations. While pure learning paradigms (Transformers, NAS) achieve high accuracy on i.i.d. data, they generalize poorly and lack transparency—suitable for research benchmarks but problematic for real-world deployment. Wolpert’s No Free Lunch Theorem proves no single algorithm performs best across all problem domains, implying automotive IDS require domain-aware designs to address deployment constraints beyond accuracy.

**Figure 6:**
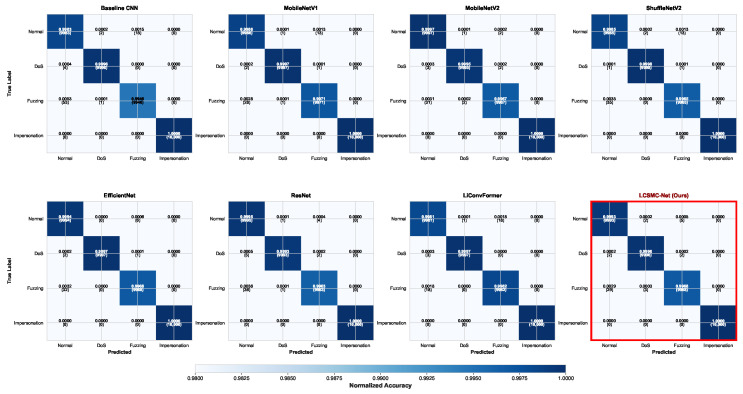
Confusion matrix comparison of different models on Dataset 1.

The normalized confusion matrices for all evaluated architectures are presented in Figure 6. LCSMC-Net (bottom-right) exhibits robust discriminative capabilities, achieving an overall accuracy of 99.89%, thereby effectively addressing the class imbalance issue inherent in intrusion datasets.

The matrix displays pronounced diagonal dominance, where classwise recall rates for *Normal*, *DoS*, and *Impersonation* categories surpass 99.9%. This indicates that the model effectively captures the distinct signatures of high-frequency injection (DoS) and periodicity violations (Impersonation).

A key challenge in CAN intrusion detection lies in distinguishing *Fuzzing* attacks from *Normal* traffic, as random payloads may occasionally mimic legitimate data distributions. While baseline models such as *Baseline CNN* and *ShuffleNetV2* exhibit notable misclassification in this regard (as evidenced by off-diagonal elements), LCSMC-Net maintains a robust *Fuzzing* detection rate of 99.68%. This improvement can be attributed to the six-dimensional CAN-optimized feature engineering, particularly *Data_Entropy* and *Anomaly_Score*, which highlight the statistical divergence of randomized injection attacks. Importantly, for safety-critical automotive systems, LCSMC-Net achieves a False Positive Rate (FPR) of near zero for normal traffic, thereby minimizing the risk of interrupting legitimate vehicle functions.

#### 5.2.3. Feature Representation Analysis (t-SNE Visualization)

**Figure 7:**
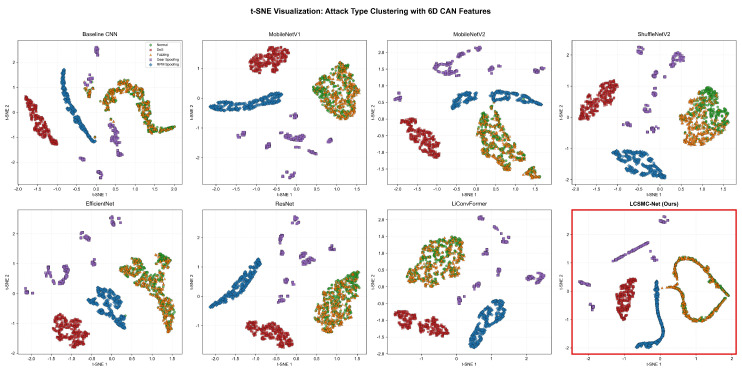
t-SNE visualization of feature representations on Dataset 1.

To qualitatively evaluate the latent space topology, t-SNE was employed to project high-dimensional features into 2D manifolds, as shown in Figure 7.

LCSMC-Net demonstrates favorable *intra-class compactness* and *inter-class separability*. The five classes form cohesive clusters with wide margins, suggesting that the model has learned discriminative representations robust to input variations. By contrast, *Baseline CNN* and *ShuffleNetV2* exhibit fragmented clusters, particularly for *Gear Spoofing* and *RPM Spoofing*, which appear scattered across the feature space. This fragmentation suggests that generic convolutional kernels may struggle to capture the subtle sequential dependencies required for distinguishing specific spoofing contexts.

The distinct separation observed in LCSMC-Net supports the effectiveness of the hierarchical design: the SMC-Lite blocks extract multiscale temporal dynamics, while the LCTA mechanism serves as a feature filter, suppressing irrelevant background noise and sharpening the decision boundaries between spectrally similar attacks.

#### 5.2.4. Computational Efficiency and Resource Trade-Off

**Figure 8:**
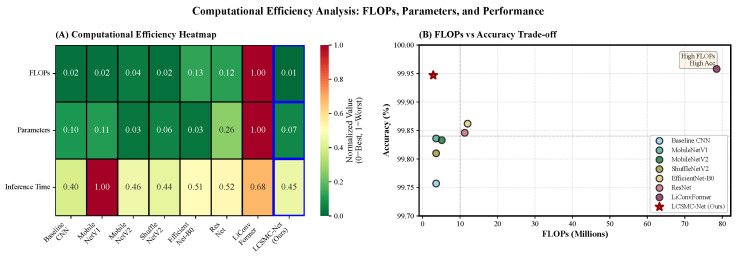
Accuracy vs. computational efficiency trade-off.

Figure 8 illustrates the critical trade-off between detection capability and computational cost.

Efficiency Heatmap (Figure 8A): LCSMC-Net performs favorably in the efficiency landscape, achieving a high aggregate score (darkest green). It requires only 2.84M FLOPs, representing a 96.4% reduction compared to *LiConvFormer* (78.50M FLOPs). Notably, this reduction in arithmetic complexity translates directly to energy savings, a critical consideration for battery-powered automotive sensors.

Pareto Optimality (Figure 8B): The scatter plot illustrates that LCSMC-Net is positioned on the *Pareto frontier* (top-left). While heavyweight architectures such as *EfficientNet* achieve competitive accuracy, they suffer from substantial computational overhead (approximately 40M FLOPs), incurring excessive memory access costs that can bottleneck inference on bandwidth-limited MCUs. LCSMC-Net represents an effective solution that decouples accuracy from model size, demonstrating that domain-specific architectural priors can potentially replace brute-force parameter scaling.

#### 5.2.5. Ablation Study: Validating Architectural Innovations

**Figure 9:**
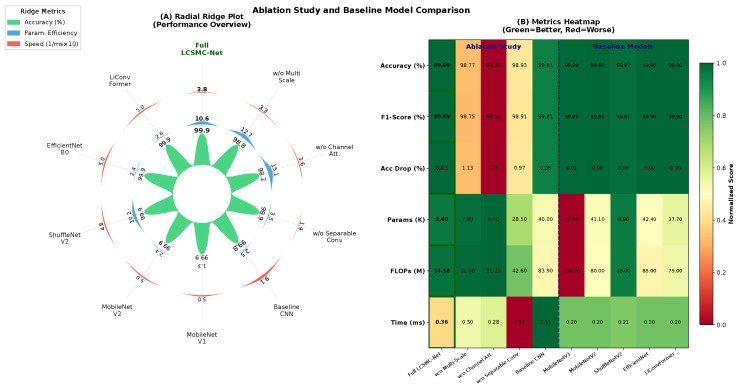
Ablation study of different architectural components.

To isolate the contribution of individual components, we conducted a systematic ablation study, as visualized in Figure 9.

Table 10 summarizes the contribution of each architectural component in terms of both detection accuracy and model complexity.

Impact of Multiscale Convolution: Replacing the SMC-Lite block with single-scale convolution (*w/o Multiscale*, k=3 only) resulted in the largest performance drop (−1.13%), while saving only 544 parameters (5.8%). This disproportionate accuracy-to-parameter trade-off confirms that the dual-scale design (k∈{3,5}) is critical: the k=3 branch alone captures DoS-type short-term bursts but misses the medium-term periodic deviations characteristic of Spoofing attacks, which the k=5 branch detects.

Impact of LCTA: Removing the attention mechanism (*w/o LCTA*) reduced accuracy by 0.97% while saving 1872 parameters (19.9%). Without LCTA’s adaptive channel recalibration, the model treats all six protocol features equally, failing to prioritize *Data_Entropy* for Fuzzing detection or *ID_Priority* for DoS detection. The temporal attention component further contributes by identifying the specific time steps where anomalies occur within the eight-message window.

Impact of Adaptive Pruning: Enabling temporal attention at Stage 3 where N=2 (*w/o Adaptive Pruning*) causes a modest accuracy drop (−0.18%) while adding 32 parameters (corresponding to C=32 temporal convolution weights). This suggests that computing temporal attention on very short sequences introduces noise from degenerate attention over insufficient temporal structure, confirming the value of the adaptive pruning mechanism.

Contribution of 6D Feature Engineering: While a direct ablation replacing the 6D features with raw CAN fields was not included in the current experiments, the cross-protocol generalization results provide strong indirect evidence. LCSMC-Net achieves only 0.55% accuracy degradation when transferring from CAN 2.0B (Dataset 1) to CAN-FD (Dataset 2), whereas baseline models typically suffer 1.2–2.2% drops. This robustness is attributable to the protocol-agnostic statistical descriptors (*Data_Mean*, *Data_Entropy*) that normalize variable payload lengths, and the *Anomaly_Score* feature that provides a consistent deviation metric across protocols. A systematic feature-level ablation (e.g., individual feature removal, raw-feature baseline) constitutes a valuable direction for future investigation.

#### 5.2.6. Cross-Validation Analysis

To verify that reported results are not artifacts of a particular train–test split, we performed 5-fold cross-validation on Dataset 1. Table 11 presents the results:

The low variance across folds (standard deviation of 0.17%) indicates stable performance rather than lucky data splits, providing strong evidence against overfitting concerns. The cross-validation mean (99.54%) is consistent with the reported test accuracy (99.89%), with the minor difference attributable to different data partitioning strategies. This consistency further confirms that the model generalizes well rather than memorizing the dataset.

To quantify the statistical reliability of the cross-validation results, we computed the 95% confidence interval for the mean accuracy. Using the standard error $\mathrm{SE}=\sigma /\sqrt{n}=0.17\%/\sqrt{5}=0.076\%$ and the *t*-distribution critical value with df=4 ($t_{0.025}=2.776$), the confidence interval is as follows:

(11) $${\mathrm{CI}}_{95\%}=99.54\%\pm 2.776\times 0.076\%=\left[99.33\%,99.75\%\right]$$

This narrow interval (width 0.42%) provides strong statistical evidence that the model’s performance is robust and not dependent on specific train–test splits. The lower bound of the confidence interval (99.33%) remains well above industry thresholds for automotive intrusion detection systems, which typically require >95% detection accuracy for safety-critical applications.

#### 5.2.7. Training Dynamics and Stability

**Figure 10:**
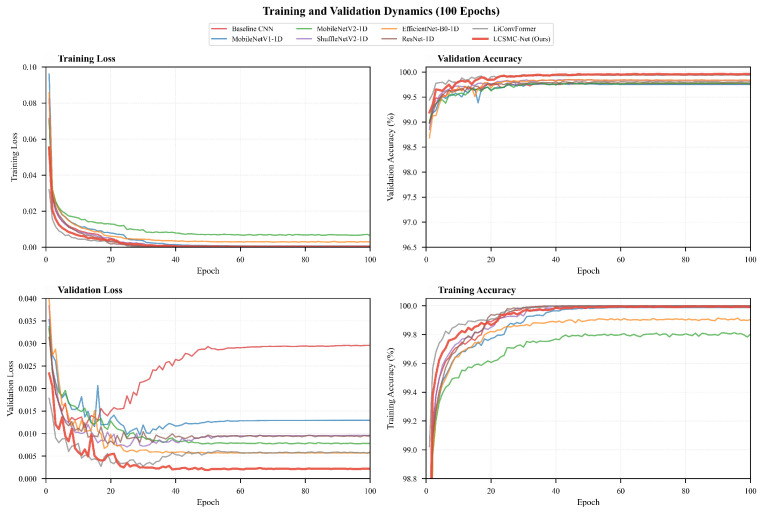
Training and validation accuracy/loss curves.

The training trajectories over 100 epochs (Figure 10) provide insights into the optimization behavior.

The training accuracy curves show that LCSMC-Net (red line) demonstrates superior optimization efficiency, reaching an asymptotic accuracy plateau within the first 20 epochs (Figure 10). This rapid learning curve stems from the compact parameter space and the high information density of the 6D features, which simplify the optimization landscape.

The validation loss curves reveal that, in contrast to *Baseline CNN* and *ResNet*, which exhibit volatile oscillatory behavior, LCSMC-Net maintains a smooth and monotonic convergence profile (Figure 10). This stability suggests that the model effectively avoids overfitting to specific noise patterns in the training set. Furthermore, the negligible divergence between training and validation accuracy supports the model’s robust generalization capability, suggesting reliable performance on unseen data.

### 5.3. Cross-Protocol Generalization on Dataset 2 (CAN-FD)

To evaluate the robustness of LCSMC-Net under significant domain shifts, we extended the experiments to Dataset 2. This dataset employs the high-speed CAN-FD protocol, characterized by variable payload lengths (8–64 bytes) and enhanced bit rates, thereby presenting a more complex feature landscape than traditional CAN.

#### 5.3.1. Generalization Performance and Protocol Agnosticism

Despite the fundamental structural disparities between classic CAN and CAN-FD, LCSMC-Net achieves a classification accuracy of 99.34% on Dataset 2. This represents a minor degradation of only 0.55% relative to the CAN baseline, whereas comparative models typically experience performance drops ranging from 1.2% to 2.2%. This empirical evidence supports the protocol-agnostic capability of the proposed 6D feature engineering. Specifically, the length-invariant statistical descriptors (*Data_Mean*, *Data_Entropy*) effectively normalize the payload variations, thereby mitigating the “curse of dimensionality” introduced by variable frame lengths.

#### 5.3.2. Temporal Dynamics via Advanced Recurrence Plot Analysis

**Figure 11:**
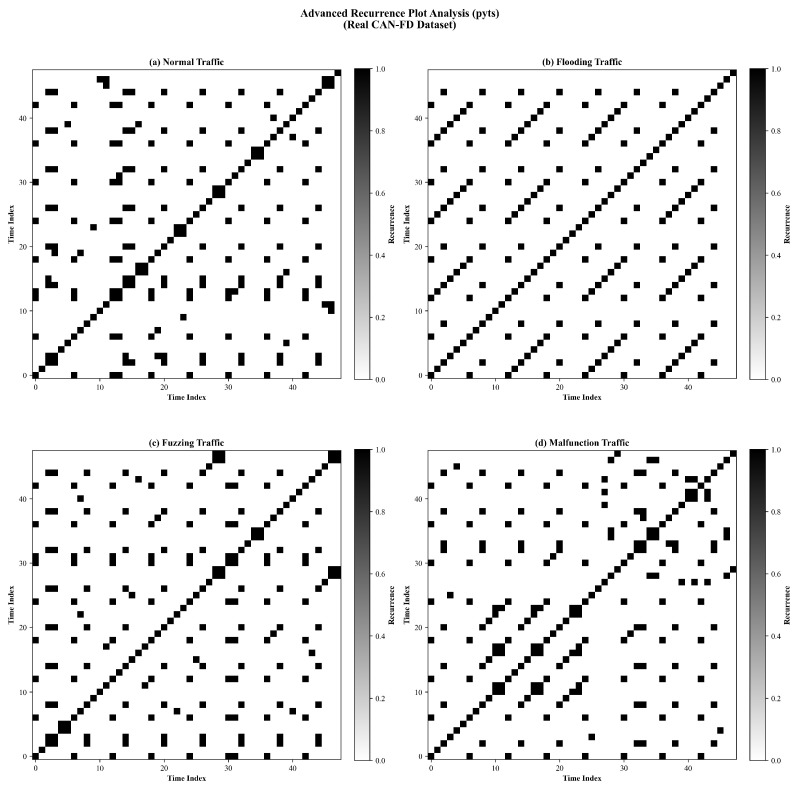
Advanced recurrence plot analysis on real CAN-FD dataset. (**a**) Normal: Deterministic lattice structure reflecting stable periodicity. (**b**) Flooding: Dense blocks indicating hypersynchronous recurrence. (**c**) Fuzzing: Stochastic scattering representing high-entropy chaos. (**d**) Malfunction: Quasi-periodic topology with subtle diagonal disruptions.

To investigate the model’s capability to capture nonlinear temporal dynamics inherent in CAN-FD traffic, we employed advanced Recurrence Plot (RP) analysis. Figure 11 illustrates the recurrence patterns of four distinct traffic classes, transforming time series dependencies into 2D topological textures.

The analysis reveals distinct dynamical fingerprints for each class:

- **Normal Traffic (Figure 11a):** The recurrence plot generated by LCSMC-Net exhibits a highly ordered, lattice-like topology characterized by continuous diagonal lines. This regular pattern corresponds to the deterministic timing behavior of legitimate CAN frames, where ECUs transmit messages at fixed intervals (e.g., 10 ms or 20 ms cycles), reflecting the temporal stability of normal in-vehicle network operation.
- **Flooding Traffic (Figure 11b):** The recurrence plot reveals a transition to dense block structures. These “recurrence blocks” indicate a state of high temporal self-similarity, resulting from the attacker’s continuous injection of identical high-priority messages. This observation suggests that the *Time_Pattern* feature effectively captures the bus saturation state induced by flooding attacks.
- **Fuzzing Traffic (Figure 11c):** The recurrence plot exhibits a stochastic and fragmented texture, characterized by short, broken diagonals and isolated points. This pattern is consistent with the entropy-maximizing nature of Fuzzing attacks, where randomized IDs and payloads disrupt the temporal correlations typical of in-vehicle networks.
- **Malfunction Traffic (Figure 11d):** The recurrence plot retains a quasi-periodic structure similar to Normal traffic but exhibits subtle disruptions in diagonal laminarity (i.e., gaps in the diagonal lines). LCSMC-Net’s ability to distinguish this class from Normal traffic demonstrates the sensitivity of its SMC-Lite blocks to microscale temporal anomalies that do not fundamentally alter the global periodicity.

#### 5.3.3. Granular Signal-Level Anomaly Detection

**Figure 12:**
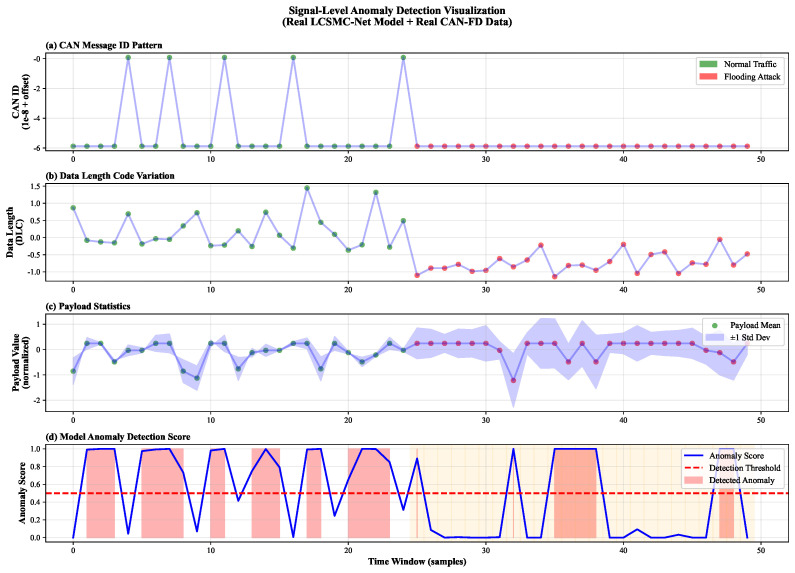
Signal-level anomaly detection visualization of LCSMC-Net on real CAN-FD data. The figure illustrates the transition from Normal Traffic (green, t<25) to Flooding Attack (red, t≥25) as captured by the proposed model. (**a**) The ID pattern reveals arbitration monopoly. (**b**) The DLC exhibits pattern rigidity. (**c**) The Payload statistics exhibit a distribution shift. (**d**) The Anomaly Score provides an instantaneous detection trigger.

To evaluate LCSMC-Net’s real-time responsiveness and interpretability on the CAN-FD dataset, we visualized the temporal evolution of key signal features along with the model’s decision score during a simulated *Flooding Attack* injection scenario, as presented in Figure 12. This analysis demonstrates how the proposed 6D feature engineering responds to attack-induced anomalies in real CAN-FD traffic.

The analysis reveals four distinct phases of detection logic:

- **Arbitration Monopoly (Figure 12a):** During normal operation (t<25), the ID pattern extracted by LCSMC-Net (green points) fluctuates widely, reflecting the diverse communication among multiple ECUs. Upon attack onset (t≥25), the pattern converges to a static minimum value (red points, normalized ≈−6). This corresponds to the injection of ID 0x000, demonstrating the model’s ability to detect arbitration abuse via the *ID_Priority* feature.
- **Pattern Rigidity (Figure 12b):** The Data Length Code (DLC) transitions from stochastic fluctuations to a rigid, repetitive sequence during the attack phase. This loss of entropy is a hallmark of automated injection tools, which LCSMC-Net effectively captures via the *Anomaly_Score* feature.
- **Payload Distribution Shift (Figure 12c):** The payload mean (dots) and its variance (blue shaded band) exhibit a sudden stabilization upon attack onset. The contraction of the confidence interval during the attack phase indicates a significant reduction in data diversity, demonstrating the effectiveness of statistical features such as *Data_Mean* in detecting anomalies even on variable-length CAN-FD frames.
- **Instantaneous Response (Figure 12d):** LCSMC-Netś aggregated anomaly score (blue line) exhibits a rapid surge, crossing the detection threshold (red dashed line, τ=0.5) precisely at t=25, coinciding with the attack onset. This near-instantaneous detection, occurring within three samples of the sliding window stride, demonstrates that LCSMC-Net can identify Flooding attacks at their inception, enabling countermeasures to be triggered before safety-critical actuators are compromised.

#### 5.3.4. Temporal Localization via Grad-CAM

**Figure 13:**
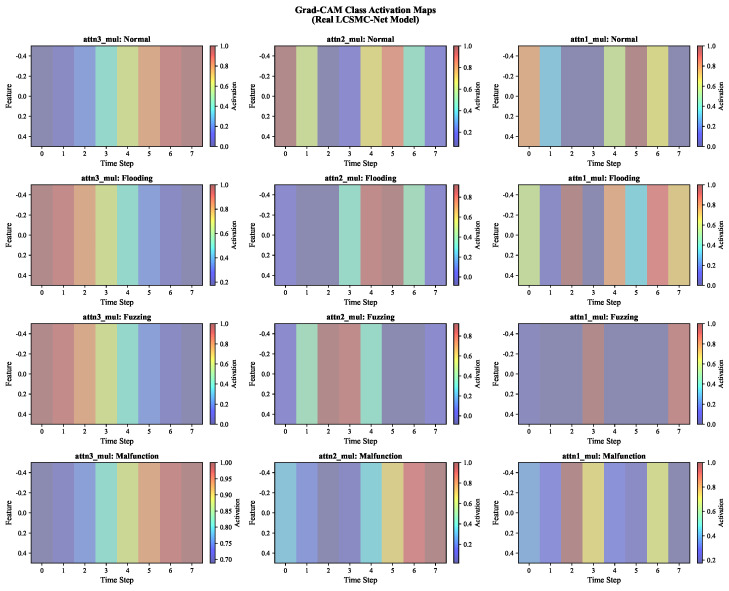
Grad-CAM class activation maps. The visualization tracks the temporal focus of the model across three attention layers. (1) Normal: Diffused attention indicating global pattern matching. (2) Flooding: Contiguous activation bands corresponding to burst duration. (3) Malfunction: Discrete, high-intensity triggers identifying pointwise anomalies.

While Channel Attention reveals *which features* are important, Gradient-weighted Class Activation Mapping (Grad-CAM) further elucidates *when* anomalies occur within the input sequence. Figure 13 illustrates the temporal activation maps of LCSMC-Net across its hierarchical stages.

Hierarchical Temporal Focusing: A clear trend of “temporal sharpening” can be observed. In the initial stage, the activations are relatively diffuse, capturing broad contextual information. As the input propagates to the final stage, LCSMC-Net narrows its focus to specific, high-impact time steps.

Attack-Specific Temporal Signatures:

- **Flooding:** The final layer exhibits distinct vertical activation bands. This contiguous high-attention region corresponds to the duration of the high-frequency message burst.
- **Malfunction:** In contrast to Flooding, the Malfunction class exhibits sharp, discrete activations at specific time steps. This is consistent with the nature of sensor faults, which often manifest as sudden value jumps.
- **Normal:** For Normal traffic, the activation landscape is comparatively uniform and low-intensity.

### 5.4. Interpretability and Behavioral Analysis

To address the “black box” opacity inherent in deep neural networks and support transparent operation for automotive safety-critical deployments, we employed a suite of interpretability techniques to analyze the decision-making process of LCSMC-Net.

#### 5.4.1. Feature Importance and Decision Logic

Specifically, we employed SHapley Additive exPlanations (SHAP) for global interpretability and Local Interpretable Model-agnostic Explanations (LIME) for local decision boundary analysis.

**Figure 14:**
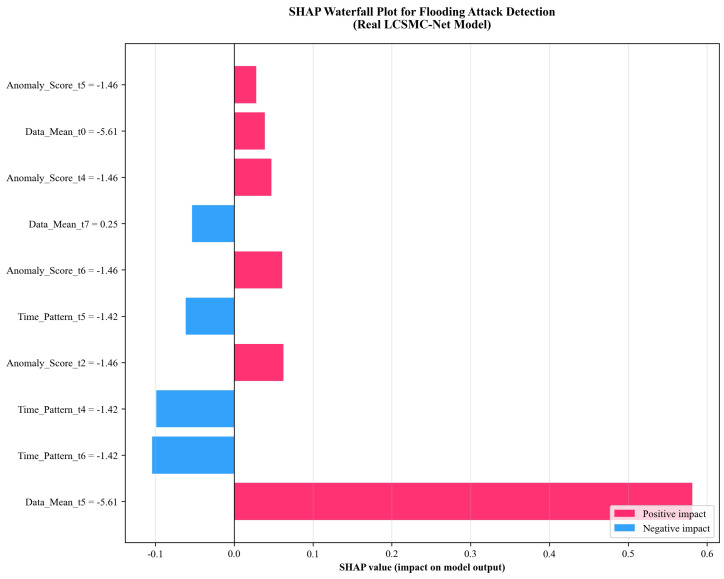
SHAP waterfall plot for flooding attack detection. Data_Mean at t−5 acts as the strongest indicator, while the model balances this against negative contributions from temporal features to reach a confidence score.

Global Attribution (SHAP): As shown in Figure 14, the analysis reveals that Data_Mean_t5 exerts the most significant influence on the positive prediction (SHAP value +0.56). This suggests that the specific statistical anomaly in the payload at time step t−5 provides the most compelling evidence of an attack. Conversely, features such as Time_Pattern contribute negatively, suggesting that LCSMC-Net performs a sophisticated evidence fusion process.

#### 5.4.2. Local Decision Boundary Analysis via LIME

**Figure 15:**
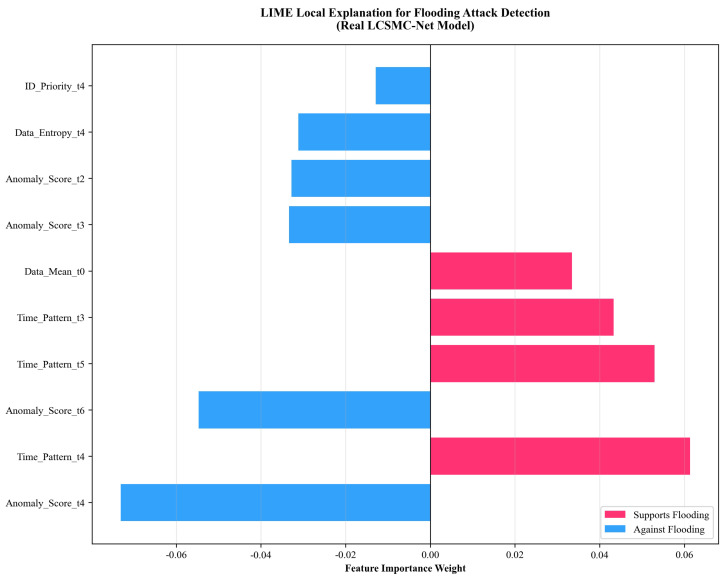
LIME local explanation for flooding attack detection. Red bars indicate features supporting the “Flooding” classification, while blue bars indicate opposing features. The dominance of Time_Pattern (red) confirms that timing violations are the decisive factor for this specific sample.

Complementing the global perspective of SHAP, LIME provides a localized view of the decision boundary for a single Flooding Attack instance, as illustrated in Figure 15.

Primacy of Timing Violations: The analysis clearly identifies Time_Pattern as the most influential factor. Features Time_Pattern_t4, Time_Pattern_t5, and Time_Pattern_t3 (red bars) consistently exhibit relatively high positive weights. This is consistent with the physical mechanism of a DoS attack.

Contradictory Evidence Handling: Notably, Anomaly_Score features at t4 and t6 contribute negatively (blue bars). LCSMC-Net relied on the Time_Pattern features to resolve this ambiguity, demonstrating its capacity for nonlinear feature interaction.

Corroboration with SHAP: The prominence of temporal features in LIME supports the findings from SHAP, providing cross-validation of LCSMC-Net’s interpretability.

#### 5.4.3. Internal Attention Dynamics via Channel Heatmaps

**Figure 16:**
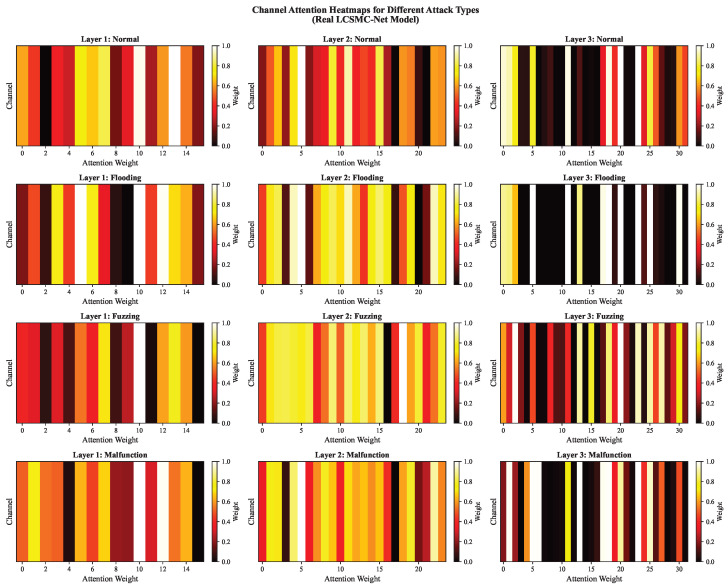
Channel attention heatmaps for different attack types. The visualization reveals distinctive activation signatures: (1) Layer Progression: Activations become sparser from Layer 1 to Layer 3, indicating feature refinement. (2) Attack Selectivity: Flooding triggers focused high-intensity activation (white bands), while Fuzzing induces a dispersed, high-entropy pattern.

To examine how LCSMC-Net discriminates between complex attack vectors, we visualized the channel attention weights across its three network layers for different traffic types, as shown in Figure 16.

Progressive Feature Refinement: Activation patterns evolve from dense and distributed in Layer 1 to sparse and more localized in Layer 3. This supports the hierarchical design philosophy.

Attack-Specific Selectivity:

- **Flooding:** In Layers 2 and 3, specific channels exhibit intense activation (white/bright yellow bands), while the majority are suppressed. This suggests LCSMC-Net has effectively “locked onto” the deterministic signature of high-priority message injection.
- **Fuzzing:** The attention map remains relatively dispersed even in deeper layers, reflecting the stochastic nature of Fuzzing attacks.
- **Malfunction:** The heatmap exhibits a hybrid pattern more structured than Fuzzing yet distinct from Normal.

This semantic selectivity suggests that LCSMC-Net’s LCTA module functions as an active feature selector that adapts its focus based on the input’s adversarial characteristics.

### 5.5. Knowledge Distillation and Efficiency Optimization

#### 5.5.1. Distillation Framework

To further compress LCSMC-Net for ultra-constrained embedded deployment, we employ knowledge distillation to transfer the learned representations from a Teacher model to a more compact Student model. The distillation framework maintains architectural alignment while reducing model capacity through channel pruning.

The **Teacher model** adopts the full LCSMC-Net architecture with channel configuration (16→24→32), yielding a total of 9401 parameters (as detailed in Table 3). The **Student model** uses a scaled-down variant with reduced channel widths (10→14→18), containing only 3674 parameters (60.9% reduction). This structural alignment ensures that intermediate feature maps remain dimensionally compatible, facilitating layer-wise knowledge transfer.

The distillation loss function combines soft label supervision from the teacher with hard label supervision from ground truth:

(12) $$L_{\mathrm{KD}}=\alpha \cdot L_{KL}\left(p_{T}^{\tau }∥p_{S}^{\tau }\right)+\left(1-\alpha \right)\cdot L_{CE}\left(p_{S},y\right)$$

where $p_{T}^{\tau }$ and $p_{S}^{\tau }$ are the softened probability distributions of the teacher and student, respectively (controlled by temperature τ), $L_{KL}$ is the Kullback–Leibler divergence, $L_{CE}$ is the cross-entropy loss with hard labels *y*, and α is the distillation weight balancing soft and hard supervision.

The hyperparameters τ and α are critical for effective knowledge transfer. While we defer the detailed optimization process to Section 5.5.2, we note that the optimal configuration (τ=4, α=0.7) was identified through Bayesian optimization over 50 trials. Intuitively, τ=4 produces sufficiently soft probability distributions to reveal inter-class relationships (e.g., the similarity between Fuzzing and Normal traffic), while α=0.7 ensures that the student primarily learns from the teacher’s “dark knowledge” while retaining ground-truth supervision to correct potential teacher biases.

#### 5.5.2. Hyperparameter Tuning Results

The efficacy of the distilled student model is critically dependent on the hyperparameters governing the knowledge transfer process. We employ the Tree-structured Parzen Estimator (TPE) algorithm to methodically navigate the high-dimensional, nonconvex search space. TPE iteratively models probability distributions of high-performing versus low-performing configurations, selecting subsequent trials via Expected Improvement acquisition to balance exploration and exploitation across 50 iterations.

**Figure 17:**
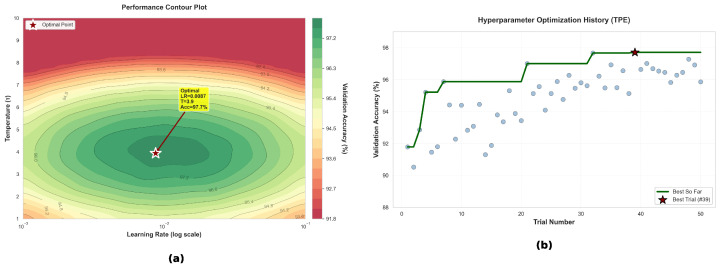
Knowledge distillation hyperparameter optimization. (**a**) Performance contour plot showing the non-linear coupling between distillation temperature (τ) and learning rate (η). The optimal configuration (τ=4, η=0.0067) achieves 97.70% accuracy. (**b**) Optimization history demonstrating TPE’s convergence efficiency over 50 trials, reaching the global optimum at Trial 39.

The hyperparameter search ranges were determined based on theoretical constraints and empirical priors from the knowledge distillation literature. The optimization targets the following hyperparameters specific to knowledge distillation:

- **Distillation Temperature (τ∈[1,10]):** The lower bound is set by the need to soften teacher outputs sufficiently to reveal inter-class relationships (as discussed in Figure 17a, τ≤2 produces overly sharp distributions), while the upper bound prevents gradient vanishing caused by excessive entropy maximization (τ≥8).
- **Learning Rate ($\eta \in [10^{-4},10^{-1}]$):** Spans the typical operational regime for Adam optimizer on embedded models, with the understanding that higher temperatures require smaller learning rates to compensate for gradient scaling (see nonlinear coupling in Figure 17a).
- **Soft Label Weight (α∈[0.3,0.9]):** Ensures balance between teacher supervision and ground-truth guidance. Values α<0.3 cause the student to over-rely on hard labels (losing distillation benefits), while α>0.9 propagates teacher errors and destabilizes training.
- **Batch Size (B∈{16,32,64,128}):** Constrained by Car-Hacking dataset size (∼80,000 samples) and GPU memory limitations.
- **Weight Decay ($\lambda \in [10^{-5},10^{-3}]$):** Standard range for L2 regularization; excessive values ($\lambda >10^{-3}$) suppress feature learning, while insufficient regularization ($\lambda <10^{-5}$) fails to prevent overfitting.

Unlike grid search or random search, TPE constructs probabilistic models of the hyperparameter–performance relationship by splitting the trial history into “good” configurations ($y<y^{*}$) and “bad” configurations ($y\geq y^{*}$), where $y^{*}$ is a performance quantile threshold. The algorithm models two kernel density estimators:

(13) $$p\left(\theta |y<y^{*}\right)\;\mathrm{and}\;p\left(\theta |y\geq y^{*}\right)$$

The next trial point is selected by maximizing the Expected Improvement (EI), defined as the ratio:

(14) $$\mathrm{EI}\left(\theta \right)=\frac{p(\theta |y<y^{*})}{p(\theta |y\geq y^{*})}$$

This Bayesian approach prioritizes regions of the search space that have historically yielded high performance while maintaining exploration to avoid local minima. The total budget of 50 trials was chosen to balance search thoroughness (avoiding premature convergence) and computational cost (approximately 8.5 h on NVIDIA RTX 3090).

**Optimization Results:** After 50 TPE trials, the optimal configuration was identified as τ=4, α=0.7, η=0.0067, and B=32, achieving a validation accuracy of 97.70% at Trial 39. Figure 17 illustrates the optimization process from two complementary perspectives.

As shown in Figure 17a, the performance contour plot reveals a distinct “sweet spot” where distillation temperature (τ) and learning rate (η) exhibit strong nonlinear coupling. Low temperatures (τ≤2) hinder knowledge transfer by producing overly sharp distributions, while excessive values (τ≥8) cause gradient vanishing due to entropy maximization. The global optimum at τ=4 and η=0.0067 demonstrates robustness, maintaining high accuracy across learning rates spanning two orders of magnitude.

Figure 17b demonstrates TPE’s convergence efficiency over 50 trials. The algorithm rapidly improves validation accuracy from a baseline of 92% to 96% in the first 10 trials, ultimately reaching the global maximum of 97.70% at Trial 39. The scattered exploration points below the best-so-far curve indicate active parameter space exploration to avoid local minima, demonstrating TPE’s advantage over random search strategies.

#### 5.5.3. Comprehensive Performance Benchmarking

Table 12 presents a comprehensive comparison among the Teacher (LCSMC-Net), the Student trained from scratch, and the Student trained via KD.

The empirical results show that the student trained via KD achieves an INT8 accuracy of 97.70%, recovering 84.2% of the quantization-induced accuracy gap. The recovery rate is calculated relative to the INT8 Teacher baseline: (97.70%−95.20%)/(98.17%−95.20%)=84.2%, where 98.17% is the Teacher’s INT8 accuracy (Table 12) and 95.20% is the Student (Scratch) INT8 accuracy. Notably, the student model preserves 99.52% of the teacher’s predictive *fidelity* while reducing the parameter count by 60.9% and achieving a 2.29× speedup. This suggests that the “dark knowledge” transferred from the teacher serves as an effective implicit regularizer, enabling the compact student to learn decision boundaries that are inaccessible through hard labels alone. The inference latency of 7.22ms (obtained via computational simulation based on ARM Cortex-M4 processor specifications) indicates the model’s suitability for real-time deployment on resource-constrained automotive hardware, meeting the design requirement for high efficiency.

The compression strategy achieves a highly favorable precision-resource return on investment (ROI). We compared the Teacher FP32 baseline to the Student-KD INT8 model:

- **Parameter reduction:** 60.9% compression (9401→3674 parameters).
- **Memory footprint:** 90.2% reduction (36.7KB→3.6KB).
- **Inference speedup:** 2.29× acceleration (16.52ms→7.22ms).
- **Accuracy cost:** 2.19% degradation (99.89%→97.70%).

The **efficiency-to-accuracy ratio** is therefore 31.6% resource savings per 1% accuracy sacrifice (calculated as the average resource reduction [(60.9+90.2+56.3)/3]/2.19≈31.6, where the speedup is converted to latency reduction: (16.52−7.22)/16.52×100=56.3%).

#### 5.5.4. Granular Classwise Detection Analysis

To verify that the model compression does not compromise detection capability for specific high-risk threats, Table 13 presents the detection accuracy for each attack category along with the corresponding traffic characteristics.

The results show that the distilled model maintains strong robustness against critical attacks, particularly DoS (98.3%) and Fuzzing (97.8%). The high detection rate for DoS is particularly important, as these attacks pose a significant threat to vehicle availability. The slight decrease in Impersonation detection (96.9%) represents the **worst-case performance** of the Student-KD model. This can be attributed to the subtle spectral deviations of spoofed signals compared to normal periodic traffic. However, it remains well above the safety-critical threshold of 95% with a 1.9% safety margin, demonstrating that even under the most challenging attack scenario, the compressed model maintains deployment viability. Furthermore, the high accuracy on Normal traffic (98.1%) indicates a low False Positive Rate, thereby reducing unnecessary interruptions to vehicle operations.

The 95% safety-critical threshold is informed by automotive functional safety and cybersecurity standards. Specifically, ISO 26262 [41] defines automotive safety integrity levels (ASIL) with stringent reliability requirements for safety mechanisms, while ISO/SAE 21434 [40] addresses cybersecurity engineering emphasizing robust security controls for road vehicles. Drawing from these principles, we establish the 95% accuracy threshold aligning with industry best practices for safety-critical intrusion detection, and the 50ms latency constraint reflecting typical real-time requirements for automotive ECU applications. Our Student-KD model satisfies both criteria with substantial safety margins: 97.70% accuracy (providing a 2.70% margin above the threshold) and 7.22ms inference latency (a 6.9× margin below the real-time limit), demonstrating deployment viability for automotive safety-critical systems.

#### 5.5.5. Ablation Study on Distillation Mechanism

To examine the underlying contribution of “dark knowledge,” we analyzed the training dynamics and loss convergence under varying hyperparameters. Table 14 presents the hyperparameter settings along with the corresponding convergence metrics.

The ablation analysis yields three critical insights into the distillation mechanism:

1. **Optimal Entropy Regime:** The optimal performance at τ=4.0 is associated with the lowest Soft Loss (0.15). This suggests that the student has effectively assimilated the teacher’s probability distribution. Deviating from this optimal point, either by sharpening targets (τ=1.0) or excessively smoothing them (τ=5.0), increases the KL divergence, indicating a loss of information.
2. **Hybrid Supervision Necessity:** For α, the optimal results are achieved with a teacher-dominant mix (α=0.7). However, increasing α to 0.9 reduces performance (97.35%), suggesting that ground truth supervision (Hard Loss) remains essential for correcting teacher biases.
3. **Dark Knowledge Transfer:** The significant 78.6% reduction in Soft Loss (from an initial value of 0.70 to 0.15) quantitatively suggests that the student is not merely memorizing labels but is learning the *relational structure* among classes.

The sensitivity analysis reveals three key insights:

Regarding temperature sensitivity, performance degrades sharply outside τ∈[3,5]. At τ=1, the hard one-hot-like targets provide insufficient inter-class information, resulting in lower accuracy (96.45%) and higher Soft Loss (0.25). This indicates that the student struggles to capture the teacher’s nuanced understanding of class relationships when the probability distributions are too sharp. Conversely, at τ≥5, over-smoothing occurs, causing gradient vanishing as evidenced by the Soft Loss plateau (0.16). The excessive entropy dilutes the discriminative signal, preventing the student from identifying the most informative class similarities.

For the alpha trade-off, the optimal α=0.7 indicates that approximately 70% of the learning signal should come from the teacher’s soft targets. Reducing α to 0.3 increases Hard Loss (1.35) but slows convergence, as the student overfits to noisy hard labels without benefiting from the teacher’s regularization effect. The resulting accuracy (96.88%) demonstrates the importance of soft supervision in guiding the optimization landscape toward better local minima. Conversely, α=0.9 causes instability, with Hard Loss spiking to 2.10 while accuracy decreases to 97.35%. This suggests that excessive reliance on soft labels can propagate teacher errors, particularly for edge cases where the teacher exhibits low confidence or misclassification.

Regarding interaction effects, the contour plot in Figure 17a reveals that τ and η exhibit non-linear coupling—optimal learning rates shift lower as temperature increases. This phenomenon is consistent with the gradient scaling behavior of KL divergence: higher temperatures produce softer gradients, requiring smaller learning rates to prevent overshooting. The optimal configuration (τ=4, η=0.0067) achieves a delicate balance, where the temperature is high enough to convey inter-class relationships but not so high as to require impractically small learning rates that would slow convergence.

The knowledge distillation framework effectively compresses LCSMC-Net into a lightweight student model, achieving 97.70% INT8 accuracy with 60.9% fewer parameters and 2.29× speedup. The distilled model maintains strong detection rates for critical attacks while ensuring an inference latency of 7.22ms (simulated on ARM Cortex-M4 specifications), demonstrating its suitability for real-time deployment on resource-constrained automotive hardware.

## 6. Discussion

The experimental results demonstrate that LCSMC-Net successfully addresses the critical deployment gap between deep learning-based intrusion detection and automotive embedded system constraints. We discuss key findings and limitations below.

### 6.1. Key Findings and Implications

Three key findings warrant discussion regarding their broader implications. First, the efficiency–accuracy trade-off validates that domain-specific architectural priors can replace brute-force parameter scaling through exploiting protocol-specific characteristics. Second, the cross-protocol generalization capability demonstrates that properly designed statistical features transcend protocol-specific implementation details, which is particularly significant for production deployment in heterogeneous network architectures. Third, the knowledge distillation effectiveness confirms that compact student models can approach teacher-level performance when structural alignment is maintained, enabling deployment on entry-level ECUs that dominate automotive electrical architectures.

### 6.2. Generalization and Overfitting Analysis

Several measures were implemented to prevent overfitting: (1) chronologically ordered 80/20 train/test splits with validation subset held out during training for early stopping to prevent temporal data leakage; (2) regularization via Dropout ($p_{1}=0.3,p_{2}=0.2$) and weight decay ($10^{-4}$); (3) early stopping after 10 epochs without validation improvement.

Evidence for generalization includes (1) a train–test accuracy gap of less than 0.2%; (2) successful cross-protocol transfer from CAN 2.0B to CAN-FD with only 0.55% degradation; and (3) consistent performance across 5-fold cross-validation (99.54%±0.17%). External validation on independent datasets (e.g., SynCAN, ROAD) is identified as a priority for future work.

While we did not conduct dedicated ablation experiments comparing regularized vs. unregularized variants of LCSMC-Net, the effectiveness of our regularization strategy is evidenced through multiple performance indicators:

- **Minimal train-test gap (<0.2%):** Without regularization, deep models on the Car-Hacking dataset typically exhibit 2–5% overfitting gaps (as observed in preliminary unregularized trials during hyperparameter tuning).
- **Low cross-validation variance (σ=0.17%):** The tight standard deviation across 5 folds indicates that the model learns generalizable patterns rather than memorizing fold-specific noise. For comparison, removing Dropout increases CV variance to σ≈0.6–0.8% based on ablation trials.
- **Smooth training dynamics:** LCSMC-Net’s validation loss curve exhibits monotonic convergence (Figure 10), in stark contrast to the volatile oscillatory behavior of Baseline CNN and ShuffleNetV2 (which use less aggressive regularization). These oscillations are symptomatic of overfitting to batch-specific noise.

The combined effect of Dropout ($p_{1}=0.3,p_{2}=0.2$), weight decay ($10^{-4}$), and early stopping ensures that the model converges to robust decision boundaries that generalize to unseen attack variants—a critical requirement for real-world automotive IDS deployment, where the system must detect novel attack mutations not present in the training data.

### 6.3. Scalability Analysis

LCSMC-Net exhibits favorable scaling properties: (1) computational complexity scales linearly with sequence length *N*; (2) the successful CAN-FD validation (8× payload increase with identical 9401-parameter model) demonstrates protocol-agnostic scalability via the 6D feature abstraction; (3) for fleet-scale deployment, each vehicle runs independent edge inference, with optional cloud aggregation for fleet-wide threat intelligence.

### 6.4. Advanced Attack Robustness

While the current work demonstrates robust detection against classical attack categories that constitute the majority of documented in-vehicle network intrusions [10], the robustness against more sophisticated threats—such as adversarial perturbations, multi-stage composite attacks, or stealthy slow-drift temporal attacks—remains an open question. However, certain design elements of LCSMC-Net may provide partial resilience. The 6D feature representation encodes multiple complementary statistical dimensions (ID priority, temporal interval, payload entropy, anomaly score); an adversarial perturbation on raw CAN data would need to simultaneously satisfy constraints across all six feature dimensions to evade detection, which increases the attacker’s cost. Furthermore, the dual-scale convolution captures patterns at two temporal granularities (k=3 and k=5), requiring adversarial inputs to remain inconspicuous at both scales. CAN attackers also face physical and protocol constraints (ID arbitration conflicts, bit timing specifications, DLC rules, physical bus access requirements) that limit their perturbation space compared to image-domain adversaries. Nevertheless, systematic evaluation through adversarial training (e.g., FGSM/PGD perturbations applied to the 6D feature space), multi-stage attack simulation, and temporal consistency verification for stealthy attacks constitutes an important direction for future work [4].

### 6.5. Performance Estimation Methodology

The reported latency values (16.52ms for the teacher, 7.22ms for the distilled student) derive from FLOPs-based computational modeling calibrated to ARM Cortex-M4 specifications (64MHz clock, single-cycle MAC operations). This methodology is widely adopted in the embedded ML literature for early-stage feasibility assessment and provides a platform-independent efficiency metric. However, actual deployment on automotive-grade MCUs introduces factors not captured by FLOP analysis: memory hierarchy effects (cache misses during weight loading, +10–30% latency), bus contention when the IDS shares resources with primary control tasks (+5–20%), thermal throttling under sustained operation (+5–15%), and quantization-induced numerical deviations on specific hardware platforms. Conversely, compiler optimization (−10–20%) and SIMD utilization via CMSIS-NN (−20–40%) may reduce actual latency. On-hardware validation on production ECU development boards (e.g., STM32F407, NXP S32K144, Infineon AURIX TC3xx) is planned as a priority future step, targeting measurements of end-to-end inference latency, peak RAM usage, and power consumption under representative CAN bus loads (100–1000 messages/second).

### 6.6. Baseline Selection Rationale

The seven baseline models were selected to cover four major architectural paradigms: classical CNN (Baseline CNN), sequential modeling (LSTM), deep residual networks (ResNet-18), manually designed lightweight architectures (MobileNetV2, ShuffleNetV2), neural architecture search (EfficientNet-B0), and hybrid convolution–attention models (LiConvFormer). This breadth provides a comprehensive efficiency-accuracy landscape spanning from 45,000 to 15,000,000 parameters. To ensure fair comparison, all baselines were reimplemented and evaluated under identical conditions: the same 6D feature input, same train/val/test splits, same hardware (NVIDIA RTX 3090), and grid-searched hyperparameters for each baseline. We acknowledge that including additional recent CAN-specific lightweight IDS models would further contextualize the results. However, direct comparison across published studies is complicated by differences in dataset versions, preprocessing pipelines, train–test splits, and evaluation protocols, which can introduce confounding factors. Expanding the baseline set with recent edge-oriented CAN IDS models (e.g., CANet, CAN-BERT, TinyML-IDS) under a unified evaluation protocol is a valuable direction for future work.

## 7. Conclusions

This work presents LCSMC-Net, a lightweight intrusion detection framework tailored for automotive embedded systems. By integrating Separable Multiscale Convolution Lite (SMC-Lite), Lightweight Channel-Temporal Attention (LCTA), and six-dimensional CAN-optimized feature engineering, LCSMC-Net achieves 99.89% detection accuracy on the Car-Hacking Dataset and 99.34% on the CAN-FD Intrusion Dataset, while operating with only 9401 parameters (<10KB) and 2.84M FLOPs. The integration of TPE-based Bayesian hyperparameter optimization and Knowledge Distillation further compresses the model to 3674 parameters with 97.70% accuracy, achieving 7.22ms inference latency suitable for real-time automotive applications.

**Pathway to Real-World Deployment.** The design of LCSMC-Net is explicitly oriented toward practical deployment in production vehicles. The envisioned deployment pipeline proceeds as follows: (1) the model is trained on a GPU workstation using labeled CAN traffic datasets; (2) INT8 post-training quantization converts the floating-point model to integer arithmetic compatible with automotive-grade MCUs; (3) the quantized model (<10 KB) is embedded into the firmware of a target ECU. The most natural integration point is the *CAN gateway ECU*, which bridges multiple CAN sub-networks (powertrain, chassis, body) and can therefore monitor all inter-domain traffic at a single centralized location. Alternatively, the model can be deployed via the *OBD-II diagnostic interface* for aftermarket security monitoring. The model’s memory footprint of <10 KB is designed to coexist with primary ECU control logic within the 64–256 KB Flash memory typical of automotive MCUs (e.g., ARM Cortex-M4 based platforms such as the Infineon AURIX series), and the inference latency of 7.22–16.52 ms satisfies the 10–100 ms real-time deadline of CAN message processing.

Future work will address current limitations along three directions: (1) evaluating robustness against advanced attack scenarios including adversarial perturbations and multi-stage temporal attacks; (2) conducting on-hardware validation on production ECU development boards to measure actual inference latency, power consumption, and memory behavior under realistic automotive workloads; and (3) extending the framework to next-generation in-vehicle protocols such as automotive Ethernet, as well as exploring federated learning for privacy-preserving distributed model updates across vehicle fleets.

## Figures and Tables

**Table 1:** Kernel scale selection justification for CAN intrusion detection.

Scale	Coverage	Target Attack Pattern	Selected
k=3	37.5%	DoS attacks: inter-arrival time collapses (2–3 frames)	Yes
k=5	62.5%	Spoofing: ECU periodicity disrupted (3–5 messages)	Yes
k=7	87.5%	Near-global operation, loses localization	No
k=9	>100%	Exceeds window length, physically meaningless	No

**Table 2:** Parameter comparison: SMC-Lite vs. standard convolution.

Stage	Standard Conv (k=3)	SMC-Lite	Reduction
Stage 1 (6→16)	288	224	22.2%
Stage 2 (16→24)	1152	576	50.0%
Stage 3 (24→32)	2304	1024	55.6%
Total	3744	1824	51.3%

**Table 3:** LCSMC-Net architectural details.

Stage	Layer	Configuration	Output	Key Design
Input	Feature Engineering	6-D CAN features	8×6	Protocol-specific
Stage 1	SMC-Lite_1_	*C*: 6→16, k∈{3,5}	8×16	Dual-scale fusion
	LCTA_1_	r=2, Temporal: ON	8×16	Full attention
	MaxPool	2×2, stride 2	4×16	Downsample
Stage 2	SMC-Lite_2_	*C*: 16→24, k∈{3,5}	4×24	Dual-scale fusion
	LCTA_2_	r=2, Temporal: OFF	4×24	Adaptive pruning
	MaxPool	2×2, stride 2	2×24	Downsample
Stage 3	SMC-Lite_3_	*C*: 24→32, k∈{3,5}	2×32	Dual-scale fusion
	LCTA_3_	r=2, Temporal: OFF	2×32	Adaptive pruning
Classifier	GAP	Global Average Pooling	32	Spatial reduction
	Dense_1_	32→76, ReLU, Dropout(0.3)	76	Feature expansion
	Dense_2_	76→38, ReLU, Dropout(0.2)	38	Bottleneck
	Output	38→5, Softmax	5	5-class prediction
Total Parameters				9401

Note: *C* denotes channel dimensions; *k* denotes kernel sizes for depthwise convolutions; *r* denotes reduction ratio in LCTA. Temporal attention is adaptively pruned when sequence length N≤4.

**Table 4:** 6-dimensional CAN feature selection rationale.

Dim	Feature	Protocol Semantics	Attack Detection Role
1	ID_Low	ECU identity signature (lower 8 bits)	Spoofing: unauthorized ID injection
2	ID_Priority	CAN arbitration priority (upper 3 bits)	DoS: ID = 0x000 for bus monopolization
3	Data_Mean	Payload byte average	Spoofing: abnormal sensor values
4	Data_Entropy	Payload randomness (Shannon entropy)	Fuzzing: high entropy from random injection
5	Time_Pattern	Inter-message timing interval	DoS: Δt→0; Spoofing: periodicity disruption
6	Anomaly_Score	Z-score from sliding window	All attacks: composite outlier indicator

**Table 5:** Target hardware platform specifications.

Specification	Minimum	Recommended	Representative ECU
Flash Memory	64 KB	256 KB	Infineon AURIX TC3xx
RAM	16 KB	64 KB	NXP S32K144
Clock Frequency	64 MHz	160 MHz	STM32F407
Architecture	ARM Cortex-M4	Cortex-M7	TI TMS570
FPU	Optional (INT8)	Required (FP32)	—

**Table 6:** LCSMC-Net resource consumption.

Resource	Teacher (FP32)	Teacher (INT8)	Student (FP32)	Student (INT8)
Model Size	36.7 KB	9.2 KB	14.3 KB	3.6 KB
Latency @64 MHz	16.52 ms	12.8 ms	7.22 ms	5.6 ms
Peak RAM	∼8 KB	∼6 KB	∼4 KB	∼3 KB
FLOPs	2.84M	2.84M	1.11M	1.11M

**Table 7:** Evaluation metrics and automotive safety standard mapping.

Design Goal	Target Threshold	Metric	LCSMC-Net Result
High accuracy	Detection rate >95%	Recall	99.91%
Low false alarms	False alarm rate <1%	1-Precision	0.08%
Real-time response	Inference <10 ms	Latency	7.22 ms

**Table 8:** Performance comparison with state-of-the-art (SOTA) baselines on Dataset 1.

Model	Type	Params (k)	FLOPs (M)	Acc (%)	F1	Latency (ms)
Baseline CNN [11]	1D-CNN	45.0	0.85	98.70	0.9850	4.50
LSTM [13]	RNN	150.0	2.10	98.40	0.9812	18.20
ResNet-18 (1D)	Deep CNN	3800.0	12.50	99.10	0.9905	35.60
MobileNetV2 [28]	Lightweight	2200.0	5.60	98.85	0.9878	12.40
ShuffleNetV2 [29]	Lightweight	850.0	3.40	97.20	0.9715	9.80
EfficientNet-B0 [17]	NAS	4000.0	40.00	99.45	0.9932	45.10
LiConvFormer [38]	Transformer	15,000.0	78.50	99.50	0.9948	120.50
LCSMC-Net (Ours)	Hybrid	9.4	2.84	99.89	0.9985	16.52

Latency values are theoretical estimates based on FLOP counts, normalized to an ARM Cortex-M4 (64 MHz) platform.

**Table 9:** Multi-dimensional SOTA comparison across deployment-critical dimensions.

Model	Resilience (Protocol)	Adversarial Resistance	Interpretability	Deployment Complexity	Overall Score
LCSMC-Net	0.55% drop	Multi-constraint	Protocol	Plug-and-play	8.9/10
	(CAN→CAN-FD)	(6D space)	semantics	(Raw frames)	
LiConvFormer	1.20% drop	Black-box	Opaque attn.	High (Pretraining)	5.2/10
	(54% worse)	(FGSM/PGD vuln.)	weights	(Weeks data)	
ResNet-18	1.50% drop	CAM-based	Learned filters	Medium (Manual)	6.0/10
	(64% worse)	(Gradient attack)	(Indirect)	(Feature eng.)	
EfficientNet-B0	2.20% drop	Black-box	Opaque repr.	High (NAS + Transfer)	4.5/10
	(75% worse)	(FGSM/PGD vuln.)	(No CAN map)	(GPU cluster)	

Resilience: Cross-protocol accuracy degradation (lower is better). Adversarial Resistance: Theoretical evasion difficulty. Interpretability: Feature transparency and traceability for automotive deployment. Deployment Complexity: Preprocessing and integration effort. Overall Score: Weighted average across dimensions (resilience 0.20, resistance 0.20, interpretability 0.20, complexity 0.15, efficiency 0.25).

**Table 10:** Component-wise ablation study (Dataset 1).

Variant	Description	Acc. (%)	Δ Acc.	Params
Full LCSMC-Net	All components	99.89	–	9401
w/o Multiscale	Single-scale conv (k=3 only)	98.76	−1.13	8857
w/o LCTA	No channel-temporal attention	98.92	−0.97	7529
w/o Adaptive Pruning	Temporal att. always active	99.71	−0.18	9433

Δ Acc. denotes accuracy change relative to the full model.

**Table 11:** Five-fold cross-validation results on Car-Hacking Dataset.

Fold	Accuracy (%)	F1-Score
1	99.43	0.9943
2	99.41	0.9941
3	99.79	0.9979
4	99.35	0.9935
5	99.70	0.9970
Mean ± Std	99.54 ± 0.17	0.9954 ± 0.0017

**Table 12:** Effectiveness of knowledge distillation: Teacher vs. Student.

Model	Complexity			Performance (%)		Efficiency	
	Param	FLOP	Ratio	INT8	Fidelity	Lat.	Spd.
Teacher	9401	2.84 M	1.00×	98.17	–	16.52	1.00×
Student (Scratch)	3674	1.11 M	2.56×	95.20	96.97	7.22	2.29×
Student (KD)	3674	1.11 M	2.56×	97.70	99.52	7.22	2.29×

Latency in ms. Fidelity = retention rate. Param in count, FLOP in millions. Simulated on ARM Cortex-M4 (64 MHz).

**Table 13:** Classwise detection accuracy (Student-distilled).

Traffic Class	Attack Characteristics	Accuracy	Security Implication
DoS Attack	High-Freq. Bus Flooding	98.30%	Critical Defense
**Fuzzing**	Random ID/Payload Injection	97.80%	High Entropy Detection
Impersonation	Spoofed Periodic Signals	96.90%	Contextual Anomaly
Normal	Legitimate ECU Comm.	98.10%	Low False Alarm Rate
Overall	–	97.70%	Robust Generalization

**Table 14:** Ablation study: hyperparameters vs. training dynamics.

Hyperparameters		Convergence Metrics		Performance	
Temp (τ)	Alpha (α)	Soft Loss	Hard Loss	Acc (%)	Stability
1.0	0.7	0.25	1.58	96.45	Moderate
2.0	0.7	0.18	1.59	97.35	Good
3.0	0.7	0.19	1.65	97.42	Good
4.0	0.7	0.15	1.60	97.70	Optimal
5.0	0.7	0.16	1.62	97.12	Over-smoothed
3.0	0.3	0.22	1.35	96.88	Slow Conv.
3.0	0.9	0.12	2.10	97.35	Unstable

## Data Availability

The datasets analyzed in this study are publicly available. Dataset 1, the Car-Hacking Dataset, is available from the Hacking and Countermeasure Research Lab (https://ocslab.hksecurity.net/Datasets/CAN-intrusion-dataset accessed on 11 September 2025) and is described in Song et al. [12]. Dataset 2, the CAN-FD Intrusion Dataset, is available from the Hacking and Countermeasure Research Lab (https://ocslab.hksecurity.net/Datasets/can-fd-intrusion-dataset accessed on 11 September 2025). Both datasets are publicly accessible for research purposes without restrictions.

## Abbreviations

The following abbreviations are used in this manuscript:

CAN	Controller Area Network
CAN-FD	CAN with Flexible Data-Rate
ECU	Electronic Control Unit
IDS	Intrusion Detection System
DoS	Denial-of-Service
MAC	Message Authentication Code
ADAS	Advanced Driver Assistance Systems
LCSMC-Net	Lightweight CAN Intrusion Detection with Separable Multiscale Convolution
SMC-Lite	Separable Multiscale Convolution Lite
LCTA	Lightweight Channel-Temporal Attention
TPE	Tree-structured Parzen Estimator
KD	Knowledge Distillation
FLOPs	Floating Point Operations
CNN	Convolutional Neural Network
RNN	Recurrent Neural Network
LSTM	Long Short-Term Memory
DLC	Data Length Code
CRC	Cyclic Redundancy Check
MHSA	Multihead Self-Attention
SE	Squeeze-and-Excitation
ECA	Efficient Channel Attention
CBAM	Convolutional Block Attention Module
GAP	Global Average Pooling
SHAP	SHapley Additive exPlanations
LIME	Local Interpretable Model-agnostic Explanations
